# Reconstructing A/B compartments as revealed by Hi-C using long-range correlations in epigenetic data

**DOI:** 10.1186/s13059-015-0741-y

**Published:** 2015-08-28

**Authors:** Jean-Philippe Fortin, Kasper D. Hansen

**Affiliations:** Department of Biostatistics, Johns Hopkins Bloomberg School of Public Health, 615 North Wolfe Road, Baltimore, 21205 MD USA; McKusick-Nathans Institute of Genetic Medicine, Johns Hopkins School of Medicine, 1900 East Monument Street, Baltimore, 21205 MD USA

**Keywords:** Methylation, Chromatin, Epigenetics, Chromosome configuration capture, Single-cell, Hi-C, DNase sequencing

## Abstract

Analysis of Hi-C data has shown that the genome can be divided into two compartments called A/B compartments. These compartments are cell-type specific and are associated with open and closed chromatin. We show that A/B compartments can reliably be estimated using epigenetic data from several different platforms: the Illumina 450 k DNA methylation microarray, DNase hypersensitivity sequencing, single-cell ATAC sequencing and single-cell whole-genome bisulfite sequencing. We do this by exploiting that the structure of long-range correlations differs between open and closed compartments. This work makes A/B compartment assignment readily available in a wide variety of cell types, including many human cancers.

**Electronic supplementary material:**

The online version of this article (doi:10.1186/s13059-015-0741-y) contains supplementary material, which is available to authorized users.

## Background

Hi-C, a method for quantifying long-range physical interactions in the genome, was introduced by Lieberman-Aiden et al. [1], and it was reviewed in Dekker et al. [2]. A Hi-C assay produces a so-called genome contact matrix, which – at a given resolution determined by sequencing depth – measures the degree of interaction between two loci in the genome. In the last 5 years, significant efforts have been made to obtain Hi-C maps at ever increasing resolutions [3–8]. Currently, the highest resolution maps are 1 kb [7]. Existing Hi-C experiments have largely been performed in cell lines or for samples where unlimited input material is available.

In Lieberman-Aiden et al. [1], it was established that at the megabase scale, the genome is divided into two compartments, called A/B compartments. Interactions between loci are largely constrained to occur between loci belonging to the same compartment. The A compartment was found to be associated with open chromatin and the B compartment with closed chromatin. Lieberman-Aiden et al. [1] also showed that these compartments are cell-type specific, but did not comprehensively describe differences between cell types across the genome. In most subsequent work using the Hi-C assay, the A/B compartments have received little attention; the focus has largely been on describing smaller domain structures using higher resolution data. Recently, it was shown that 36 % of the genome changes compartment during mammalian development [8] and that these compartment changes are associated with gene expression; they conclude “that the A and B compartments have a contributory but not deterministic role in determining cell-type-specific patterns of gene expression”.

The A/B compartments are estimated by an eigenvector analysis of the genome contact matrix after normalization by the observed–expected method [1]. Specifically, boundary changes between the two compartments occur where the entries of the first eigenvector change sign. The observed–expected method normalizes bands of the genome contact matrix by dividing by their mean. This effectively standardizes interactions between two loci separated by a given distance by the average interaction between all loci separated by the same amount. It is critical that the genome contact matrix is normalized in this way, for the first eigenvector to yield the A/B compartments.

Open and closed chromatin can be defined in different ways using different assays such as DNase hypersensitivity or chromatin immunoprecipitation (ChIP) sequencing for various histone modifications. While Lieberman-Aiden et al. [1] established that the A compartment is associated with open chromatin profiles from various assays, including DNase hypersensitivity, it was not determined to what degree these different data types measure the same underlying phenomena, including whether the domain boundaries estimated using different assays coincide genome-wide.

In this manuscript, we show that we can reliably estimate A/B compartments as defined using Hi-C data by using Illumina 450 k DNA methylation microarray data [9] as well as DNase hypersensitivity sequencing [10, 11], single-cell whole-genome bisulfite sequencing (scWGBS) [12] and single-cell assay for transposase-accessible chromatin (scATAC) sequencing [13]. Data from the first two assays are widely available for a large number of cell types. In particular, the 450 k array has been used to profile a large number of primary samples, including many human cancers; more than 20,000 samples are readily available through the Gene Expression Omnibus (GEO) and The Cancer Genome Atlas (TCGA) [14]. We show that our methods can recover cell-type differences. This work makes it possible to study A/B compartments comprehensively across many cell types, including primary samples, and to investigate further the relationship between genome compartmentalization and transcriptional activity or other functional readouts.

As an application, we show how the somatic mutation rate in prostate adenocarcinoma (PRAD) is different between compartments and we show how the A/B compartments change between several human cancers; currently TCGA does not include assays measuring chromatin accessibility. Furthermore, our work reveals unappreciated aspects of the structure of long-range correlations in DNA methylation and DNase hypersensitivity data. Specifically, we observe that both DNA methylation and the DNase signal are highly correlated between distant loci, provided that the two loci are both in the closed compartment.

## Results and discussion

### A/B compartments are highly reproducible and are cell-type specific

**Fig. 1:**
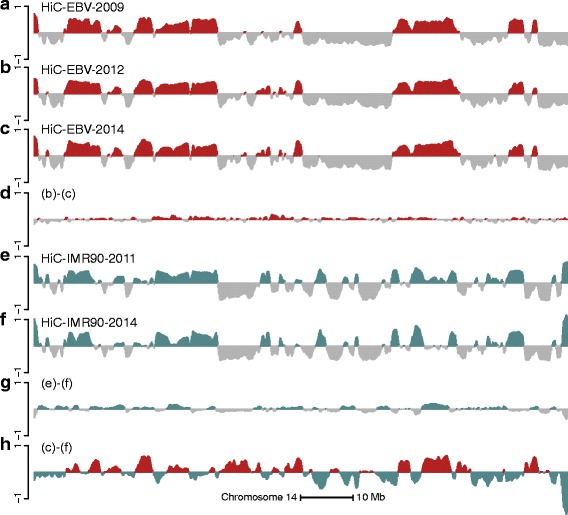
A/B compartments are reproducible and cell-type specific. The figure displays data on all of chromosome 14 at 100-kb resolution. The first eigenvector is shown for the observed–expected normalized (**a**) HiC-EBV-2009, (**b**) HiC-EBV-2012 and (**c**) HiC-EBV-2014 datasets. **d** The difference between (**b**) and (**c**). The first eigenvector is shown for the observed–expected normalized (**e**) HiC-IMR90-2013 and (**f**) HiC-IMR90-2014 datasets, and (**g**) their difference. **h** The difference between (**c**) and (**f**), which is greater than the technical variation depicted in (**d**) and (**g**). This establishes that Hi-C compartments are highly reproducible between experiments in different laboratories and that compartments are cell-type specific

We obtained publicly available Hi-C data on Epstein–Barr virus (EBV)-transformed lymphoblastoid cell lines (LCLs) and fibroblast cell lines and estimated A/B compartments by an eigenvector analysis of the normalized Hi-C contact matrix (“Materials and methods”). The contact matrices were preprocessed with iterative correction and eigenvector decomposition (ICE) [15] and normalized using the observed–expected method [1]. As in Lieberman-Aiden et al. [1], we found that the eigenvector divides the genome into two compartments based on the sign of its entries. These two compartments have previously been found to be associated with open and closed chromatin; in the following, we will use open to refer to the A compartment and closed to refer to the B compartment. The sign of the eigenvector is arbitrary; in this manuscript, we select the sign so that positive values are associated with the closed compartment (“Materials and methods”). In Fig. 1, we show estimated eigenvectors at 100-kb resolution from chromosome 14 across two cell types measured in multiple laboratories with widely different sequencing depth, as well as variations in the experimental protocol. We observed a very high degree of correspondence between replicates of the same cell type; on chromosome 14, the correlation between eigenvectors from experiments with the same cell type is greater than 0.96 (ranges from 0.96 to 0.98). The agreement, defined as the percentage of genomic bins that are assigned to the same compartment in two different experiments, is greater than 92 *%* (ranges from 92.6 % to 96.0 %) on chromosome 14. These measures vary little between chromosomes; a full depiction is available in Additional file 1: Figure S1.

Using high-resolution data does not change the estimated A/B compartments as seen in Additional file 1: Figure S2. Note that the Hi-C datasets have been processed into unadjusted contact matrices using different alignment and filtering pipelines (see “Materials and methods” for details); this shows that the choice of alignment and filtering method has negligible impact on estimation of A/B compartments.

Figure 1 shows the A/B compartments are cell-type specific, with a variation between cell types that exceeds technical variation in the assay; this has been previously noted [1, 8]. The correlation between eigenvectors from different cell types is around 0.60, in contrast to 0.96+ between eigenvectors from the same cell type.

ICE normalization removes any marginal dependence of the contact matrix on GC content by forcing the marginal sums of the contact matrix to be constant [15]. Despite this, Imakaev et al. [15] found high correlation (0.80) between the first eigenvector of the contact matrix and GC content of the underlying bin, and interpreted this as a biological association and not technical bias. To investigate further whether this dependence is a result of technical bias or a biological association, we computed the dependence for multiple experiments (Additional file 1: Figure S3). Like the eigenvector itself, we found that the dependence shows little variation between experiments done on the same cell line but in different labs, and some variation between cell lines (Additional file 1: Figures S3 and S4). This comparison includes two cell line experiments performed in the same laboratory with the same experimental protocol. That the effect of GC content depends on the cell line suggests that the relationship at least partly reflects biology. Various biological entities are correlated with GC content, including gene density [16]; it is therefore not inconceivable that open and closed chromatin has a biological association with GC content. It is possible computationally to adjust for the dependence on GC content by regressing out the fitted LOESS curve displayed in Additional file 1: Figure S3; like Imakaev et al. [15], we currently believe that doing so will remove some biological signals.

In the remainder of the manuscript, we use the most recent data, i.e. HiC-EBV-2014 and HiC-IMR90-2014, to represent eigenvectors and A/B compartments derived from Hi-C data in these cell types.

### Predicting A/B compartments from DNA methylation data

To estimate A/B compartments using epigenetic data other than Hi-C, we first concentrate on DNA methylation data assayed using the Illumina 450 k microarray platform. Data from this platform are widely available across many different primary cell types. To compare with existing Hi-C maps, we obtained data from 288 EBV-transformed LCLs from the HapMap project [17].

**Fig. 2:**
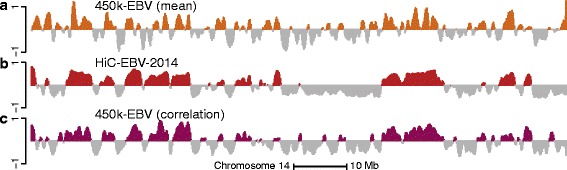
The methylation correlation signal is a better predictor of A/B compartments than the average methylation signal. The figure displays data on all of chromosome 14 at 100-kb resolution. **a** The smoothed, average methylation signal on the beta-value scale for the 450 k-EBV dataset. The signal has been centered by the mean and the sign has been reversed so that values close to one correspond to low methylation values. **b** The first eigenvector of the HiC-EBV-2014 Hi-C dataset. **c** The smoothed first eigenvector of the binned correlation matrix of the 450 k-EBV dataset. We see that (**c**) correlates better with (**b**) than (**a**)

DNA methylation is often described as related to active and inactive parts of the genome. Most established is high methylation in a genic promoter leading to silencing of the gene [18]. As a first attempt to predict A/B compartments from DNA methylation data, we binned the genome and averaged methylation values across samples and CpGs inside each bin. Only CpGs more than 4 kb away from CpG islands were used; these are termed open sea CpGs (“Materials and methods”). We found that high levels of average methylation were associated with the open compartment and not the closed compartment; this might be a consequence of averaging over open sea probes. Figure 2 depicts data from such an analysis for LCLs on chromosome 14 at a 100-kb resolution. It shows there is some agreement between estimated compartments from Hi-C and this analysis, with a correlation of 0.56 and a compartment agreement between datasets of 71.7 *%* on this chromosome. In this analysis, we implicitly assume that there is no variation in compartments between different individuals for the same cell type.

Surprisingly, we found that we could improve considerably on this analysis by doing an eigenvector analysis of a suitably processed between-CpG correlation matrix (Fig. 2). This matrix represents correlations between any two CpGs measured on the 450 k array, with the correlation being based on biological replicates of the same cell type. The correlation eigenvector shows strong agreement with the Hi-C eigenvector, certainly higher than with the average methylation vector (Fig. 2). Quantifying this agreement, we found that the correlation between the two vectors is 0.85 and the compartment agreement is 83.8 % on chromosome 14. Genome-wide, the correlation is 0.71 and the agreement is 79 % (Table 1); chromosome-specific measures are depicted in Additional file 1: Figure S5; we tend to perform worse on smaller chromosomes. Again, this analysis implicitly assumes lack of variation in compartments between biological replicates.

**Table 1:** Correlation and agreement between Hi-C and 450 k-based eigenvector estimates of genome compartments. Thresholding refers to excluding genomic bins where the entries of the relevant eigenvector have an absolute value less than 0.01

		Chromosome 14		Genome	
No threshold					
	Correlation	0.85	0.75	0.70	0.74
	Agreement	83.7 %	79.1 %	79.0 %	79.5 %
	Bins retained	100 %	100 %	100 %	100 %
Threshold, methylation					
	Correlation	0.87	0.78	0.74	0.77
	Agreement	88.8 %	83.4 %	87.9 %	87.9 %
	Bins retained	86 %	85 %	78 %	79 %
Threshold, methylation and Hi-C					
	Correlation	0.89	0.84	0.77	0.81
	Agreement	93.0 %	90.5 %	92.6 %	92.8 %
	Bins retained	76 %	67 %	66 %	64 %

Closely examining differences between the 450 k-based predictions and the Hi-C-based estimates, we found that almost all disagreements between the two methods occur when an entry in one of the two eigenvectors is close to zero; in other words, where there is uncertainty about the compartment in either one of the two analyses. Excluding bins where the 450 k-based prediction is close to zero, that is bins that have an absolute eigenvector value less than 0.01, we got an agreement of 88.8 *%* (14.2 *%* of the bins excluded). Excluding bins where either the 450 k-based prediction is close to zero or the Hi-C eigenvector is close to zero, we got an agreement of 93 *%* (24.8 *%* of the bins excluded).

Our processing of the correlation matrix is as follows (see “Materials and methods” for details); the rationale behind our choices will be explained later in the manuscript. First, in our correlation matrix, we only included so-called open sea CpGs; these CpGs are more than 4 kb away from CpG islands. Next, we binned each chromosome into 100-kb bins and computed which open sea CpGs are inside each bin; this varies between bins due to the design of the 450 k microarray. To get a single number representing the correlation between two bins, we took the median of the correlations of the individual CpGs located in each bin. We obtained the first eigenvector of this binned correlation matrix and gently smoothed the signal by using two iterations of a moving average with a window size of three bins.

The sign of the eigenvector is chosen so that the sign of the correlation between the eigenvector and column sums of the correlation matrix is positive; this ensures that positive values of the eigenvector are associated with the closed compartment (see “Materials and methods”).

### Long-range correlations in DNA methylation data predict A/B compartment changes between cell types

**Fig. 3:**
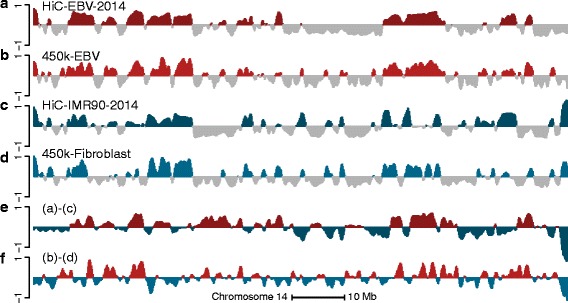
Cell-type-specific A/B compartments using Hi-C data are predicted using DNA methylation data. The figure displays data on all of chromosome 14 at 100-kb resolution. **a** The first eigenvector of the HiC-EBV-2014 dataset. **b** The smoothed first eigenvector of the binned correlation matrix of the 450 k-EBV dataset. **c** The first eigenvector of the HiC-IMR90-2014 Hi-C dataset. **d** The smoothed first eigenvector of the binned correlation matrix of the 450 k-fibroblast dataset. **e** The difference between (**a**) and (**c**). **f** the difference between (**b**) and (**d**). The high correlation between (**e**) and (**f**) supports that the correlation eigenvectors of the 450 k data can be used to find differences between compartments in the two cell types

To examine how well the predictions based on long-range correlations in 450 k data capture differences between cell types, we obtained publicly available 450 k data from 62 fibroblast samples [19], and compared them to Hi-C data from the IMR90 cell lines. Note that the fibroblast cell lines assayed on the 450 k platform are from primary skin in contrast to the IMR90 cell line, which is a fetal lung fibroblast. Figure 3, Table 1 and Additional file 1: Figure S5 show our ability to recover the A/B compartments in fibroblasts; it is similar to our performance for EBV-transformed lymphocytes.

To establish firmly that the high correlation between our predicted compartments using DNA methylation and Hi-C data is not due to chance, we compared the predicted compartments in EBV-transformed lymphocytes and fibroblasts to Hi-C data from different cell types, including the K562 cell line, which serves as a somewhat independent negative control. In Additional file 1: Figure S6, we show the correlation and agreement between the two sets of predicted compartments and Hi-C data from the three cell types. There is always a decent agreement between predicted compartments of any two cell types, but the agreement is consistently higher when the prediction is from data from the same cell type, such as the Hi-C data.

How to quantify best the differences in A/B compartments is still an open question. Lieberman-Aiden et al. [1] used 0 as a threshold to differentiate the two compartments. Considering the difference of two eigenvectors derived in different cell types, it is not clear that functional differences exist exactly when the two eigenvectors have opposite signs; instead, functional differences might be associated with changes in the magnitude of the eigenvectors reflecting a genomic region being relatively more open or closed. We note that the genomic region highlighted as cell-type specific, and validated by fluorescence in situ hybridization, in Lieberman-Aiden et al. [1], is far away from zero in one condition and has small values fluctuating around zero in the other condition.

Following this discussion, we focus on estimating the direction of change in eigenvectors between different cell types. Figure 3 shows estimated differences between Hi-C and 450 k eigenvectors for two cell types. Large differences between the two vectors are replicated well between the two data types, but there is disagreement when the eigenvectors are close to zero. This is to be expected; there is technical variation in such a difference even between Hi-C experiments (Fig. 1). Using the data displayed in Fig. 1, we found that the technical variation in the Hi-C data is such that 98 % of genomic bins have an absolute value less than 0.02. Using this cutoff for technical variation, we found that the correlation between the two difference vectors displayed in Fig. 3 is 0.85 when restricted to the 24 % of genomic bins where both vectors have an absolute value greater than 0.02. The signs of the differential vectors are also in high agreement; they agree in 90 % of the genomic bins exceeding the cutoff for technical variation. In contrast, the correlation is 0.61 when the entire chromosome is included, reflecting that the technical noise is less correlated than the signal.

Large domains of intermediate methylation have been previously described [20], as well as long blocks of hypomethylation associated with colon cancer and EBV transformation [21–23]. We obtained previously characterized [20] partially methylated domains (PMDs) in IMR90 and found a significant overlap with closed compartments from the HiC-IMR90-2014 dataset (odds ratio: 13.6) as well as closed compartments from the 450 k-fibroblast dataset (odds ratio: 16.4). Likewise, we obtained previously characterized blocks of hypomethylation associated with EBV transformation [23] and found a significant overlap with closed compartments from the HiC-EBV-2014 dataset (odds ratio: 11.9) and 450 k-EBV dataset (odds ratio: 9.4). This confirms the overlap, previously described by Berman et al. [21], between Hi-C compartments and these types of methylation domain.

### The structure of long-range correlations in DNA methylation data

To understand why we are able to predict open and closed compartments using the 450 k array, we studied the structure of long-range correlations in DNA methylation data. First, we noted that entries in our binned correlation matrix (within a chromosome) do not decay with distance between bins (Additional file 1: Figure S7a). This is in contrast to a Hi-C contact matrix, which has repeatedly been shown to decay with distance as expected (Additional file 1: Figure S7b). However, for the first eigenvector to define open and closed compartments, the Hi-C contact matrix needs to be normalized using the observed–expected method [1]. This normalization has the consequence that values in the matrix no longer decay with distance (Additional file 1: Figure S7c).

**Fig. 4:**
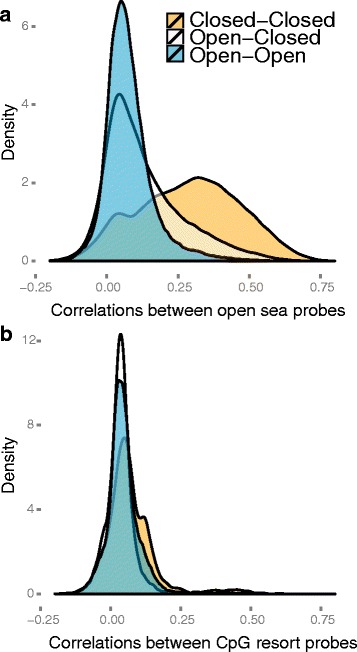
Densities of the correlations of the 450 k methylation probes. Chromosome 14 was binned at resolution 100 kb and we display the binned, stratified correlations for the 450 k-EBV dataset. Each plot shows one density curve for each type of interaction: between two bins in open compartments, between two bins in closed compartments and between a bin in the open compartment and the closed compartment. **a** Binned correlations for open sea probes only. **b** Binned correlations for CpG resort probes only. Most correlations are around zero, except correlations between two open sea probes in the closed compartment. The open and closed compartments were defined using the HiC-EBV-2014 dataset

**Fig. 5:**
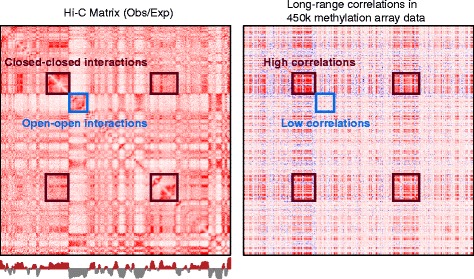
The relationship between a Hi-C contact matrix and a binned DNA methylation correlation matrix. Depicted are the observed–expected normalized genome contact matrix for the HiC-IMR90-2014 dataset together with the binned correlation matrix for the 450 k-fibroblast dataset. Both matrices depict chromosome 14 at resolution 100 kb. There is a relationship between A/B compartments in the Hi-C data and regions with low and high correlations

In Fig. 4, we show density plots of binned correlations on chromosome 14, stratified in two ways. The first stratification separates correlations between bins that are both in the open compartment or both in the closed compartment, and also cross-compartment correlations. This stratification shows that we have a large number of intermediate correlation values (0.2–0.5), but only between bins that are both in the closed compartment. The second stratification separates open sea probes and CpG resort probes (probes within 4 kb of a CpG island; see “Materials and methods”). This stratification shows that we only have intermediate correlation values for open sea probes; CpG resort probes are generally uncorrelated. In conclusion, we have the following structure of the binned correlation matrix: most of the matrix contains correlation values around zero (slightly positive), except between two bins both in the closed compartment, which have an intermediate correlation value of 0.2–0.5. This shows why an eigen analysis of the binned correlation matrix recovers the open and closed compartments; see Fig. 5 for an illustration.

The lack of decay of correlation with distance extends even to trans-chromosomal correlations, again with a clear difference between correlations within the open compartment and the closed compartment (Additional file 1: Figure S8).

**Fig. 6:**
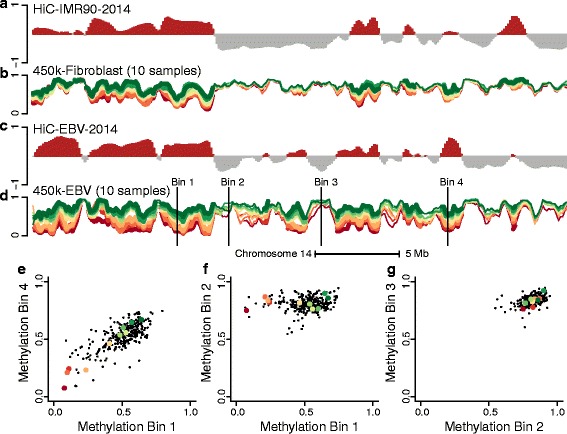
Comparison of the methylation levels and the Hi-C compartment signal for chromosome 14. The figure displays data from 36.4 to 69.8 Mb on chromosome 14 at 100-kb resolution. **a** The first eigenvector from the HiC-IMR90-2014 dataset. **b** Average methylation on the beta scale for ten selected samples from the 450 k-fibroblast dataset; each sample is a line and divergent colors are used to distinguish the different levels of methylation in the different samples. **c** The first eigenvector from the HiC-EBV-2014 data. **d** Like (**b**), but for ten samples from the 450 k-EBV dataset; the samples from the two datasets are unrelated. On (**d**) we depict four different bins. Scatterplots between methylation values in different bins across all samples in the dataset are shown in (**e**–**g**). **e** For two bins in the closed compartment. **g** For one bin in the open and one bin in the closed compartment. **g** For two bins in the open compartment. The figure shows that samples have roughly the same ranking inside each closed compartment

To understand what drives the correlation between loci within the closed compartment, we carefully examined the DNA methylation data in these genomic regions. Figure 6 shows a very surprising feature of the data, which explains the long-range correlations. In this figure, we have arbitrarily selected ten samples and we plot their methylation levels across a small part of chromosome 14, with each sample having its own color. Data from both EBV-transformed lymphocytes and fibroblasts are depicted. While the same coloring scheme has been used for both cell types, there is no correspondence between the samples assayed in the different experiments. The figure shows that the ten samples have roughly the same ranking inside each region in the closed compartment. This illustrates a surprising genome-wide ranking between samples in the closed compartment.

To gain more insights into whether this ranking is caused by technical artifacts or whether it reflects real differences between the biological replicates, we obtained data where the exact same HapMap samples were profiled in two different experiments using the Illumina 27 k methylation array. This array design is concentrated around CpG islands, but we determined that 5599 probes are part of the 450 k array and annotated as open sea probes. For these probes, we determined which were part of the closed compartment and we computed the sample-specific average methylation in this compartment as a proxy for the observed ranking described above. In Additional file 1: Figure S9a, we show that the genome-wide correlation of these measurements between hybridization duplicates from the same experiment is high (0.927). In Additional file 1: Figure S9b, we show that these measurements replicate well between different experiments (correlation of 0.744).

**Fig. 7:**
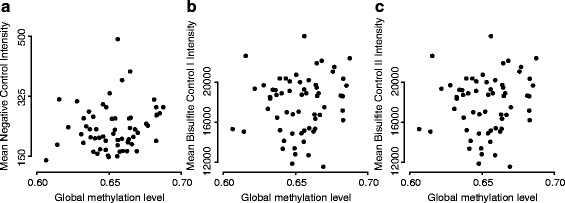
Mean methylation levels in the 450 k-fibroblast dataset are not associated with technical control probes. For each of the 62 samples from the 450 k-fibroblast dataset, we computed the average methylation level for the open sea probes and looked for association with the array technical control probes. **a** Average intensity of the negative control probes against the average methylation level. **b** Same as (**a**) but for bisulfite conversion efficiency control probes Infinium I. **c** Same as (**b**) but for bisulfite conversion efficiency control probes Infinium II. We conclude that the average methylation levels are not associated with known technical covariates

For the 450 k-fibroblast experiment, we had access to the raw IDAT files and therefore to the control probes located on the array. For this dataset, we examined if the striking global ranking between different samples using the open sea probes in the closed compartment could be explained by technical factors such as bisulfite conversion. To test this, we regressed the mean (and median) methylation levels against each of the following five variables: chip and well variables (surrogates for batch), Bisulfite I and Bisulfite II control probes and negative control probes (background noise). None of these variables was significantly associated with the mean of the median methylation levels (all *P* values greater than 0.09 and *R*^2^ less than 16 %); see Fig. 7. We conclude that the global ranking cannot be explained by technical issues.

Finally, using the 27 k data, we show that the eigenvector replicates between a 450 k experiment and a 27 k experiment using the same cell type (EBV) but different samples (correlation of 0.89; see Additional file 1: Figure S10). As a control, we compared with a 450 k-derived eigenvector for a different cell type (fibroblast) and observed weak correlation (0.40). We note that the eigenvector derived from the 27 k experiment is based on far fewer probes; we do not recommend using 27 k data to estimate compartments. This result shows that the estimated genome compartments do not depend on the design of the microarray and suggests that our observations are common across methylation assays.

### The impact of GC content on long-range correlations in DNA methylation data

**Fig. 8:**
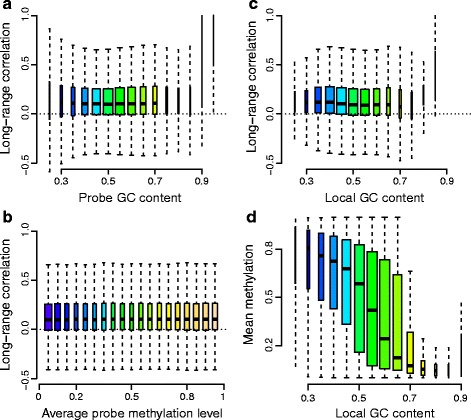
Relationship between long-range correlations, GC content and methylation levels for the 450 k-EBV dataset. Only open sea probes were included in the analysis. **a** Long-range correlations of the methylation levels stratified by probe GC content. **b** Same as (**a**), but GC content was measured in a 1-kb window around the probe. **c** Long-range correlations of the methylation levels stratified by average probe methylation. **d** Relationship between mean methylation level and GC content. While regions with high GC content tend to have low methylation, for instance CpG islands, we do not observe any relationship between the GC content of the open sea probes and the long-range correlations. We conclude that GC content is not a bias of our methylation correlation analysis

To examine the impact of GC content on the distribution of correlations, we computed this distribution as a function of both the GC content of the probe and a 1-kb window around the probe (Fig. 8a, b), and did not observe any dependence of the distribution of probe-specific correlations on GC content. The same was true when we examined the distribution of correlations as a function of the methylation level of the probe (Fig. 8c). This is in sharp contrast to the well-known high degree of association between methylation and GC content in 1 kb around the probe (Fig. 8d). In Fig. 8, we have only displayed open sea probes, and we note that these probes cover a wide range of GC content and methylation values. These results strongly suggest that the low correlations observed for CpG resort probes are not a technical artifact caused by GC content or probe-level methylation.

Because the Hi-C based eigenvectors are associated with GC content, it is expected to see such an association for 450 k-derived eigenvectors. To estimate how much of the correlation between Hi-C and methylation is due to GC content, we applied a GC content stratified permutation procedure similar to what Imakaev et al. [15] used. Briefly, we sorted the Hi-C and methylation eigenvectors by GC content and permuted neighbors within a five-bin window (to keep GC content roughly unchanged) and recalculated the correlation between the two eigenvectors. We generated 100 such permutations. While the genome-wide correlation between the Hi-C and methylation eigenvectors is high before permutation (0.74), the correlation drops to 0.21 after permuting (0.20 and 0.22 for the 2.5 and 97.5 percentiles, respectively); see Table 2 as well as Table 3 for domain agreements. We conclude that GC content by itself fails to explain the high correlation between the Hi-C and methylation eigenvectors. Based on these results, and the reasoning above, we caution that removing the GC content effect might remove a biological signal. Nevertheless, we examined whether adjusting for GC content in both Hi-C and 450 k eigenvectors would change the association between the two vectors. Before LOESS correction, the genome-wide correlation between the two eigenvectors for the EBV data is 0.71 with a domain agreement of 79 %. After GC content adjustment, the residual eigenvectors are still highly correlated (0.69) with a domain agreement of 77 %; see Additional file 1: Figure S11. This shows that adjusting for GC content does not diminish our ability to estimate A/B compartments using 450 k methylation data.

**Table 2:** Genome-wide eigenvector correlations before and after permutation

	Original	Permuted
	correlation	correlation (CI)
HiC-EBV-2014 vs. HiC-EBV-2013	0.97	0.39 (0.38–0.40)
HiC-IMR90-2014 vs. HiC-IMR90-2013	0.98	0.23 (0.21–0.24)
HiC-EBV-2014 vs. 450 k-EBV	0.73	0.21 (0.20–0.22)
HiC-IMR90-2014 vs. 450 k-fibroblast	0.75	0.18 (0.17–0.19)
HiC-EBV-2014 vs. DNase-EBV	0.71	0.41 (0.40–0.42)
HiC-IMR90-2014 vs. DNase-IMR90	0.78	0.22 (0.21–0.23)

CI: confidence interval

**Table 3:** Genome-wide domain agreements before and after permutation

	Original	Permuted
	agreement %	agreement % (CI)
HiC-EBV-2014 vs. HiC-EBV-2013	93.3	66.6 (66.1–67.2)
HiC-IMR90-2014 vs. HiC-IMR90-2013	94.5	59.0 (58.5–59.6)
HiC-EBV-2014 vs. 450 k-EBV	79.1	58.9 (58.4–59.5)
HiC-IMR90-2014 vs. 450 k-fibroblast	79.5	56.6 (56.2–57.2)
HiC-EBV-2014 vs. DNase-EBV	78.9	66.8 (66.3–67.3)
HiC-IMR90-2014 vs. DNase-IMR90	81.3	58.6 (58.0–59.1)

CI: confidence interval

### Sometimes compartment prediction fails using DNA methylation data

**Fig. 9:**
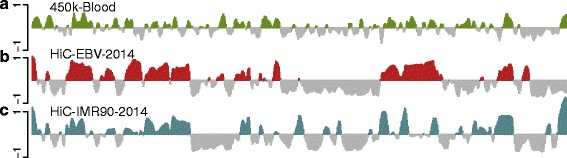
The methylation correlation signal of the 450 k-blood dataset does not correlate well with other datasets. The figure displays data on all of chromosome 14 at 100-kb resolution. **a** The smoothed first eigenvector of the binned correlation matrix of the 450 k-blood dataset. **b** The first eigenvector of the HiC-EBV-2014 dataset. **c** The first eigenvector of the HiC-IMR90-2014 dataset. We see that (**c**) does not correlate well with (**b**) and (**a**)

**Fig. 10:**
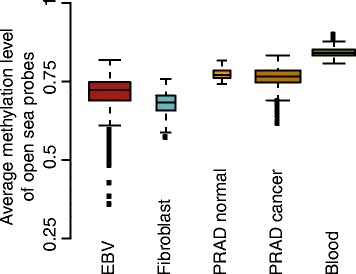
Between-sample variability in marginal methylation. For each dataset, the box plot shows the distribution of average methylation levels of the open sea probes on the beta-value scale. We are able to estimate compartments for all datasets except the 450 k-blood dataset

We caution that it is not always possible to estimate A/B compartments using data from the 450 k DNA methylation array. As an example, we present an analysis of 305 whole blood samples described previously [24]. The first eigenvector from this dataset is shown in Fig. 9. It is immediately clear that this eigenvector looks different from the other datasets we present; it seems to be oscillating more rapidly. While compartments are cell-type specific, in our experience compartments from any two cell types are somewhat correlated, reflecting that large parts of the genome do not change compartment. For example, the correlation between HiC-EBV-2014 and HiC-IMR90-2014 is 0.66 with a domain agreement of 73.4 %. In contrast, this 450 k dataset from whole blood has a correlation and domain agreement of 0.27 and 59.7 % with HiC-EBV-2014 and 0.27 and 59.6 % with HiC-IMR90-2014. The data were quantile normalized and adjusted for cell-type composition as described in [24], but we also obtained and preprocessed the raw data to exclude that data processing was the cause of the poor performance. We note that the percentage variance explained by the first eigenvector was only 57 %, in contrast to 85 % for the 450 k-EBV dataset and 74 % for the 450 k-fibroblast dataset. Based on our insights above, we hypothesized that the poor performance might be related to the lack of between-sample variability in marginal methylation, as shown in Fig. 10. However, one dataset on primary prostate shows a similar degree of between-sample variability in marginal methylation and our method works for this dataset (see below).

### Notes on processing of the DNA methylation data

We have analyzed a wide variety of DNA methylation data both from the Illumina 450 k and Illumina 27 k microarrays. For each dataset, which kind of data is publicly available varies (raw or processed). If possible, we have preferred to process the data ourselves starting from the Illumina IDAT files. However, for several datasets, we had to use the original authors’ preprocessing pipeline; see “Materials and methods” for details.

We examined the impact of preprocessing methods on the estimated eigenvectors by using functional normalization [25], quantile normalization adapted to the 450 k array [26] and raw (no) normalization; we did not find any substantial changes in the results. The agreement between the eigenvectors using the different preprocessing methods is greater than 94 % and we note that the agreement with Hi-C data is best using functional normalization. This might be caused by the ability of functional normalization to preserve large differences in methylation between samples [25], which is what we observe in the closed compartment.

We examined the binning resolution of our approach using data from the 450 k methylation array. As resolution increases, the number of bins with zero or few probes per bin increases. In Additional file 1: Figure S12, we show the trade-off between bins with zero probes and agreement with Hi-C data. This figure shows that a reasonable lower limit of resolution is 100 kb. We note that the compartments estimated from Hi-C data do not change with increased resolution (Additional file 1: Figure S2).

### An application to prostate cancer

**Fig. 11:**
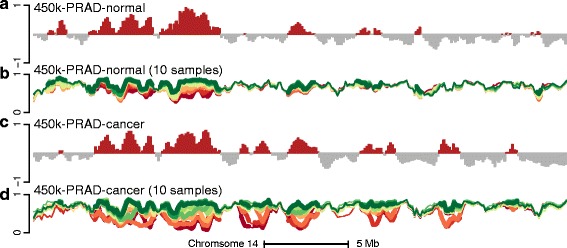
Comparison of the methylation levels and the Hi-C compartment signal for the 450 k-PRAD datasets. As Fig. 6, but for the 450 k-PRAD-cancer/normal datasets. **a** The first eigenvector of the binned methylation correlation matrix for the 450 k-PRAD-normal dataset. **b** Average methylation signal on the beta scale for ten selected samples for the 450 k-PRAD-normal dataset. **c** Like (**a**) but for the 450 k-PRAD-cancer dataset. **d** Like (**b**) but for the 450 k-PRAD-cancer dataset

We applied these methods to Illumina 450 k data on PRAD from TCGA. Quality control shows both normal and cancer samples to be of good quality. Since the normal prostate samples represent uncultured primary samples, we confirmed that this dataset has the same information in its long-range correlation structure as established above (Fig. 11; compare with Fig. 6).

**Fig. 12:**
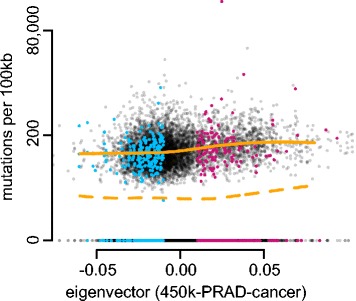
Relationship between A/B compartments and somatic mutation rate in prostate cancer. Somatic mutation rate for prostate cancer calculated using whole exome sequencing data from TCGA displayed against the first eigenvector of the 450 k-PRAD-cancer dataset. The *y*-axis uses the hyperbolic arcsine scale, which is similar to the logarithm for values greater than 1. A large number of genomic bins have a mutation rate of zero. The *dashed orange line* is a LOESS curve fitted to all the data and the *orange line* is a LOESS curve fitted only to bins with a strictly positive mutation rate. We observe an increase in somatic mutation rate in the closed compartment, as expected. *Colored points* represent bins that confidently change compartments between normal samples and cancer samples; *blue* is closed to open and *red* is open to closed. A bin confidently changes compartment if its associated eigenvector value has a magnitude greater than 0.01 (but with different signs) in both datasets

We obtained a list of curated somatic mutations from TCGA and used them to compute simple estimates of the somatic mutation rate in each 100-kb bin of the genome (i.e. the elevated mutation rate in the cancer samples compared to normals). Since the list of somatic mutations was obtained using whole-exome sequencing, we identified the capture assay used in these experiments and used the capture regions from this specific assay to compute somatic mutation rates for each 100-kb genomic bin by computing the number of somatic mutations per base captured in that bin. Because the capture assay is biased towards coding regions, the somatic mutation rates we computed can roughly be interpreted as the somatic mutation rate in coding regions per genomic bin. Many genomic bins have a somatic mutation rate of zero, and the number of bases captured varies between bins. In Fig. 12, we display this somatic mutation rate vs. the value of the first eigenvector of the cancer data. In this figure, we display two smoothed LOESS curves; one curve includes bins with a mutation rate of zero, the other excludes them. Both curves show an elevated somatic mutation rate in the closed compartment of the cancer samples. This confirms previous observations about the relationship between mutation rates and open and closed chromatin [27], including cancer [28, 29]. To our knowledge, this is the first time a cancer-specific map of open and closed compartments based on primary samples has been derived; existing analyses depend on chromatin assays performed for Encyclopedia of DNA Elements (ENCODE) and Epigenomics Roadmap samples [28, 29].

While open and closed chromatin are cell-type specific, it is not surprising that a large percentage of the genome (74 %) is in the same compartment in both normal and cancer samples. To illustrate the added value of a cancer-specific map of open and closed chromatin, we focused on the somatic mutation rate of bins that change compartment between normal and cancer. These bins are displayed in color in Fig. 12. In Table 4, we computed the average somatic mutation rate across these bins. First, as shown above, the somatic mutation rate across the the part of the genome that is open in both cancer and normal was 54.1 compared to 97.2 for the part of the genome that is closed in both cancer and normals. Focusing on the parts of the genome that change compartments, we observed that the somatic mutation rate in the parts of the genome that change from closed to open in cancer was 58.0, close to the somatic mutation rate of 54.1 in the open compartment. Conversely, the somatic mutation rate for the parts of the genome changing from open to closed in cancer was 83.9, closer to the somatic mutation rate of 97.2 in the closed compartment. This result suggests that the somatic mutation rate of a genomic region that changes compartment depends only on the compartment status of the cancer samples. One possible explanation for this, is that changes in chromatin accessibility happen relatively early in cancer development and that such changes affect the somatic mutation rate; this is highly speculative. Our result illustrates the added value of obtaining cancer-specific maps of open and closed chromatin.

**Table 4:** Number of somatic mutations per 100 kb in PRAD stratified by compartment

	Normal	
Cancer	Open	Closed
Open	54.1	58.0
Closed	83.9	97.2

### Compartments across human cancers

**Fig. 13:**
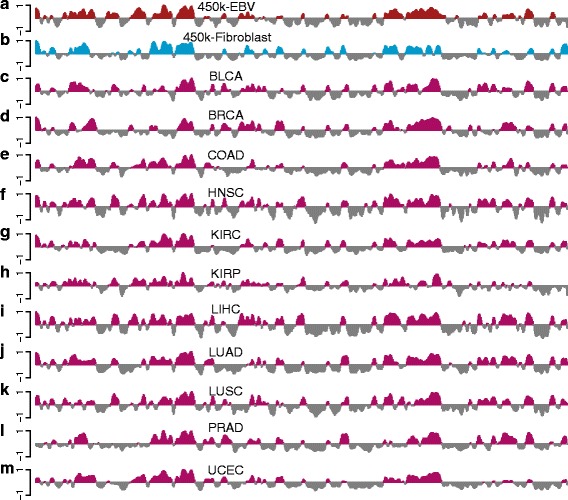
Estimated A/B compartments across several human cancers. The figure displays data on all of chromosome 14 at 100-kb resolution. Each track represents the first eigenvector of the methylation correlation matrix for the corresponding dataset. The datasets depicted in (**a**) and (**b**) are the 450 k-EBV and 450 k-fibroblast datasets. The datasets in (c–m) are cancer samples from TCGA for different cancers: (**c**) bladder urothelial carcinoma (BLCA), (**d**) breast invasive carcinoma (BRCA), (**e**) colon adenocarcinoma (COAD), (**f**) head and neck squamous cell carcinoma (HNSC), (**g**) kidney renal clear cell carcinoma (KIRC), (**h**) kidney renal papillary cell carcinoma (KIRP), (**i**) liver hepatocellular carcinoma (LIHC), (**j**) lung adenocarcinoma (LUAD), (**k**) lung squamous cell carcinoma (LUSC), (**l**) prostate adenocarcinoma (PRAD), and (**m**) uterine corpus endometrial carcinoma (UCEC)

Using the method we have developed in this manuscript, it is straightforward to estimate A/B compartments across a wide variety of human cancers using data from TCGA. Figure 13 displays the smoothed first eigenvectors for chromosome 14 at 100-kb resolution for 11 different cancers. Regions of similarity and differences are readily observed. We emphasize that TCGA does not include assays measuring chromatin accessibility such as DNase or various histone modifications. The extent to which these differences are associated with functional differences between these cancers is left for future work. Estimated compartments for all these cancer datasets are available online (see “Materials and methods”).

### Compartment prediction using DNase hypersensitivity data

Lieberman-Aiden et al. [1] established a connection between A/B compartments and DNase data, mostly illustrated by selected loci. Based on these results, we examined the degree to which we can predict A/B compartments using DNase hypersensitivity data. These data, while widely available from resources such as ENCODE, do not encompass as wide a variety of primary samples as the Illumina 450 k methylation array.

We obtained DNase sequencing (seq) data on 70 samples [30] from EBV-transformed lymphocytes from the HapMap project, as well as four experiments on the IMR90 cell line performed as part of the Roadmap Epigenomics project [31]. We computed coverage vectors for each sample and adjusted them for library size.

**Fig. 14:**
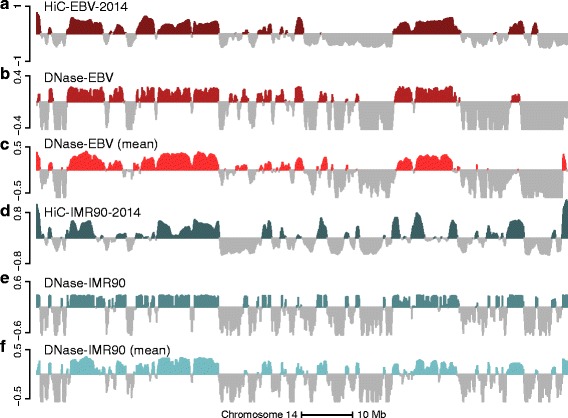
DNase data can predict A/B compartments revealed by Hi-C. The figure displays data on all of chromosome 14 at 100-kb resolution. **a** The first eigenvector of the HiC-EBV-2014 dataset. **b** The smoothed first eigenvector of the correlation matrix of the binned DNase-EBV dataset after median centering. **c** Average DNase signal across samples after binning and median subtraction. The sign of the signal was reversed for display purposes. **d** The first eigenvector of the HiC-IMR90-2014 dataset. **e** The smoothed first eigenvector of the correlation matrix of the binned HiC-DNase-IMR90 dataset after median centering. **f** Average DNase signal across samples after binning and median subtraction. The sign of the signal was reversed for display purposes. Both the average signal and correlation eigenvector are highly predictive of the Hi-C compartments for both cell types

For each sample, we computed the signal in each 100-kb genomic bin. To obtain the average DNase signal, we averaged the signal across samples. The resulting mean signal is skewed towards positive values in the open compartment, and we therefore centered the signal by the median. The median was chosen as this has the best compartment agreement with Hi-C data. Figure 14 shows the result of this procedure, slightly modified for display purposes (the sign was changed to let high values be associated with the closed compartment; additionally very low values were thresholded). A good visual agreement is observed for both cell types; the correlation between Hi-C and the average DNase signal on chromosome 14 is 0.68 for EBV and 0.75 for IMR90 with a compartment agreement of 82 % for EBV and 82 % for IMR90.

Inspired by the success of considering long-range correlations for the 450 k data, we examined whether this approach is useful for DNase data. We therefore computed the Pearson correlation matrix of the binned DNase signal; in contrast to the 450 k data, we did not bin the correlation matrix as the signal matrix was already binned. The first eigenvector of this correlation matrix is highly skewed; we centered it by its median. Figure 14 shows the result of this procedure. For chromosome 14, we obtained a correlation between this centered eigenvector and the Hi-C eigenvector of 0.75 for EBV and 0.76 for IMR90 and a compartment agreement of 86 % for EBV and 80 % for IMR90; Additional file 1: Figure S13 depicts these measures for additional chromosomes. These results are similar to what we obtained using the average DNase signal.

We observed an association between GC content and average DNase signal (Additional file 1: Figure S14); this is expected. There is a small between-sample variation in GC content effect. It is easy to remove this GC content effect by estimating the effect of using LOESS and subsequently regressing it out. Doing so led to much worse results when estimating compartments using the average DNase signal, but the results obtained using our correlation method were only slightly negatively impacted. To be precise, for the average DNase signal on chromosome 14, we got a correlation 0.35 for EBV and 0.69 for IMR90 with a compartment agreement of 69 % for EBV and 78 % for IMR90. For our correlation-based method, we got a correlation of 0.68 for EBV and 0.78 for IMR90 and a compartment agreement of 78 % for EBV and 81 % for IMR90.

**Fig. 15:**
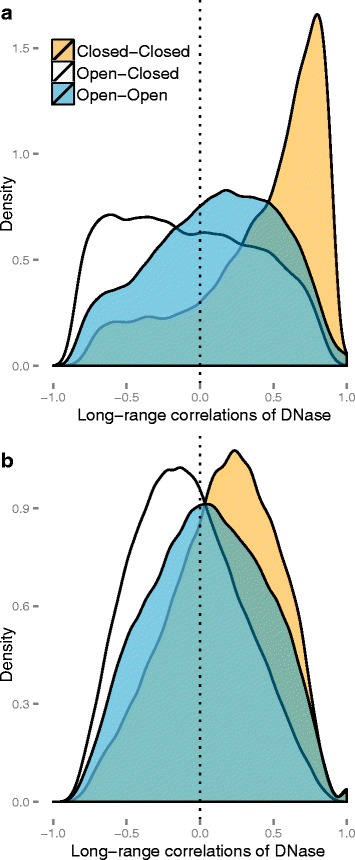
Densities of the correlations of DNase data. Chromosome 14 was binned at resolution 100 kb. Depicted are the correlations of these data for the DNase-EBV dataset, stratified by compartment type. The open and closed compartments were defined using the HiC-EBV-2014 dataset. **a** The correlations without GC content correction. **b** The correlations after GC content correction. This figure is similar to Fig. 4

To examine why the correlation-based approach works for DNase data, we performed the same investigation as for the 450 k datasets. In Fig. 15, we show the distribution of correlations stratified by compartment type. As for the DNA methylation data, the DNase data have high positive correlations between bins in the closed compartment, although the correlations in the DNase data are much higher. For DNA methylation data, correlations were close to zero between loci when at least one locus was in the open compartment. In contrast, the DNase data show an almost uniform distribution of correlation values when one of the two loci are in the open compartment. In the same figure, we display the distribution of correlations when we used a sample-specific GC content effect correction; this correction changes the correlation substantially and suggests that some of the correlation structure is driven by GC content. Nevertheless, correcting for this effect slightly decreased our power to estimate the Hi-C compartments.

Above, we have examined correcting for a sample-specific GC content effect. It is also possible directly to regress out the effect of GC content on the estimated eigenvector. Doing so, on both DNase and Hi-C data, does not decrease the correlation between the two eigenvectors (Additional file 1: Figure S13). As discussed earlier in this manuscript, we do not recommend doing this, as we believe it might remove a biological signal.

### Compartment prediction using single-cell epigenetic data

Experimental techniques for measuring epigenetics in a single cell are in rapid development. We have applied our methods to data from the few genome-wide, single-cell epigenetic experiments available. This includes data on both chromatin accessibility [13] and DNA methylation [12].

**Fig. 16:**
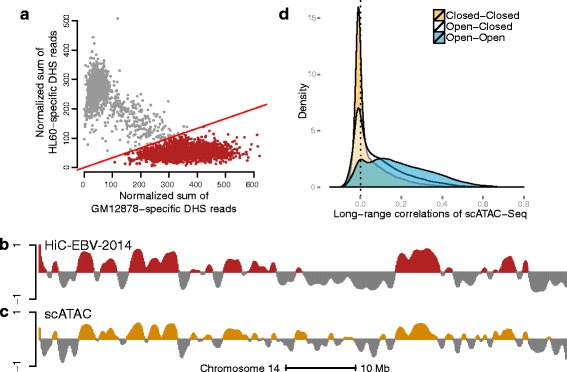
scATAC-seq data. Data from a single experiment on a mixture of the GM12878 and HL60 cell lines described in [13]. **a** ENCODE DNAse-seq data were used to define hypersensitive sites (DHSs) specific to these two cell lines. For each of these two sets of sites, we computed the average number of ATAC-seq reads normalized by the total number of reads mapped to known DHS sites. The figure shows two distinct clusters; we arbitrarily selected the line *y*=*x*/3 to delineate cells from the GM12878 cell line (*red points*); this defines the scATAC-EBV data containing 2677 cells. **b** Estimated compartments on chromosome 14 at a resolution of 100 kb using the HiC-EBV-2014 data. **c** Estimated compartments for the scATAC-EBV data. **d** Density of correlations for scATAC-EBV. We observe that the three different types of correlations have different distributions. Closed–closed correlations are skewed towards negative values, while open–open correlations are shifted towards positive values

Chromatin accessibility is measured by a single-cell variant of an assay called assay for transposase-accessible chromatin (ATAC) sequencing [32], which generates data similar to DNase hypersensitivity. From Cusanovich et al. [13], data are available on mixtures of two cell lines, GM12878 and HL60, but not on pure samples of one cell type. First, we developed a simple method for assigning single cells from this mixture to one of the two known cell lines, based on average accessibility of known cell-type-specific hypersensitive sites; this is a much more simple method than what is suggested in Cusanovich et al. [13]. Using our method, we observed two distinct clusters of cells, and most cells can easily be assigned unambiguously to a cell type using an arbitrary but seemingly sensible cutoff (“Materials and methods,” Fig. 16a). This yielded data on 2677 cells from the GM12878 cell line from one experiment. We next applied our correlation-based approach to these data; now the correlation is between single cells within the same cell line. Furthermore, the data consist of accessibility quantified over 195,882 hypersensitive sites the original authors derived from ENCODE data, with the accessibility of each site being a value of 0, 1 or 2. We summarized these data in 100-kb bins (see “Materials and methods”), not unlike our treatment of bulk DNase-seq data. On chromosome 14, we observed a correlation of 0.84 and a compartment agreement of 81 % between the first eigenvector of these data and the first eigenvector from HiC-EBV-2014 data (Fig. 16b, c). We observed that the three different types of correlations have different distributions, very different from other data types (Fig. 16d). Closed–closed correlations are skewed towards negative values, while open–open correlations are shifted towards positive values.

**Fig. 17:**
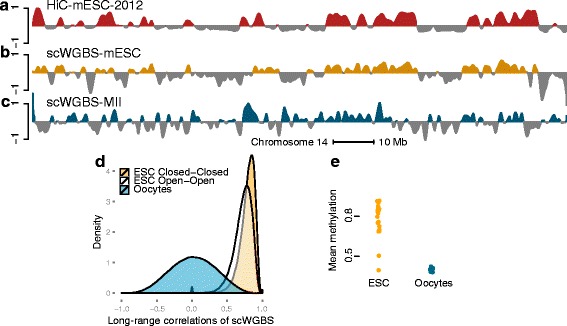
scWGBS data. Depicted are data from experiments on mESCs. **a** Estimated compartments using the HiC-mESC-2012 data on chromosome 12 at a resolution of 100 kb. **b** Estimated compartments using scWGBS data from 20 mESCs grown on serum. **c** The first eigenvector of a correlation matrix obtained using scWGBS data from 12 ovulated metaphase II (MII) oocytes. **d** Density of correlations for data on mESCs and MII cells; compartments are estimated using the HiC-mESC-2012 data. The two cell types have very different patterns. **e** Genome-wide methylation for 20 mESCs and 12 ovulated MII oocytes. Substantial heterogeneity is observed for the former but not the latter

Single-cell DNA methylation can be measured using a form of whole-genome bisulfite sequencing (WGBS) as described in Smallwood et al. [12]. Due to technical limitations of the assay, the number of assayed cells is small. We have data on 20 individual mouse embryonic stem cells (mESCs) cultured in serum conditions, with corresponding Hi-C data from a different source [3]. We generated a binned methylation matrix by averaging methylation values for open sea CpGs and discarded bins with little or no data (see “Materials and methods”). We next applied our correlation-based approach to these data, computing a correlation matrix across these 20 cells. On mouse chromosome 12, we observed a correlation of 0.61 and a domain agreement of 81 %, using existing Hi-C data on the mESC line J1 [3] (Fig. 17a–c). An analysis of the pattern of correlation between loci in open and closed compartments showed some differences between the two distributions (Fig. 17d), although both open–open and closed–closed are highly correlated in contrast to other data types. In contrast to what we observed for 450 k data, loci in the open domain are still substantially positively correlated. We note that [12] show substantial between-cell heterogeneity in genome-wide methylation across these 20 cells, depicted in Fig. 17e. However, this heterogeneity of genome-wide methylation was not observed for mouse ovulated metaphase II (MII) oocytes (Fig. 17e); the correlation distribution is substantially different for this dataset (Fig. 17d) and the first eigenvector of the correlation matrix only explains 19 % of the variance, in contrast to 99 % of the variance explained for mESCs (Fig. 17c). We do not have Hi-C data available for this cell type, but based on these observations we are doubtful that the first eigenvector accurately reflects the A/B compartments in this cell type.

## Conclusions

In this work, we show how to estimate A/B compartments using long-range correlations of epigenetic data. We have comprehensively evaluated the use of data from the Illumina 450 k DNA methylation microarray for this purpose; such data are widely available on many primary cell types. Using data from this platform, we can reliably estimate A/B compartments in different cell types, as well as changes between cell types.

This result is possible because of the structure of long-range correlations in this type of data. Specifically, we found that correlations are high between two loci both in the closed compartment and low otherwise, and do not decay with distance between loci. This result only holds true for array probes measuring CpGs located more than 4 kb from CpG islands, so-called open sea probes. This high correlation is the consequence of a surprising ranking of DNA methylation in different samples across all regions belonging to the closed compartment. We have replicated this result in an independent experiment using the Illumina 27 k DNA methylation microarray.

We have furthermore established that A/B compartments can be estimated using data from DNase hypersensitivity sequencing. This can be done in two ways: first by simply computing the average DNase signal in a genomic region, and second by considering long-range correlations in the data, like for 450 k array data. Again, we exploited the structure of long-range correlations in this type of epigenetic data and, as for DNA methylation data, we found that correlations between loci both in the closed compartment are high, whereas correlations between other loci are approximately uniformly distributed. Again, this correlation is caused by a ranking of the DNase signal in different samples across all regions belonging to the closed compartment. Surprisingly, our method works both for biological replicates (EBV-transformed lymphocytes) but also on technical between-lab replicates of the same cell line (IMR90).

Finally, we have established that our method works on single-cell epigenetic data, including scATAC-seq and scWGBS. These experimental techniques are in their infancy; it is likely that additional data will allow us to tune aspects of our method to this type of data. Now, the correlation is between single cells as opposed to biological replicates of bulk cells. This potentially allows our method to be used on rare types of cells. During the review of this paper, Buenrostro et al. [33] appeared in press, with the same conclusion as ours: scATAC-seq can reveal features of the Hi-C contact matrix.

Recently, clusters of DNA methylation under genetic control (GeMes) have been described [24]. These clusters of highly correlated CpGs are different from the compartments described here. This work described 2100 such clusters in whole blood ranging in size from 6 to 50 bp. Only five of these are greater than 10 kb and 1953 are smaller than 1 kb.

Our approach is based on computing the first eigenvector of a (possibly binned) correlation matrix. It is well known that this eigenvector is equal to the first left-singular vector from the singular value decomposition of the data matrix. The right-singular vector of this matrix is in turn equal to the first eigenvector of the sample correlation matrix, also called the first principal component. This vector has been shown to carry fundamental information about batch effects [34]. Because of this relationship, we are concerned that our method might fail when applied to experiments that are heavily affected by batch effects; we recommend careful quality control of this issue before further analysis.

We have examined the impact of GC content on our method. It has previously been established that GC content is associated with A/B compartments [15]. This association can be removed computationally but we, and Imakaev et al. [15], are concerned that it might remove a biological signal. Nevertheless, our correlation-based method shows good agreement between compartments estimated using Hi-C data and estimated using other epigenetic data, whether or not the GC content effect is removed. We have also established that GC content itself is not the main driver of long-range correlations.

The reason our method works is a surprising, consistent ranking of different samples across all regions belonging to the closed compartment (and only the closed compartment). By comparison with additional 27 k methylation array experiments, we have shown that this ranking is not a technical artifact caused by (for example) hybridization conditions.

We caution that while we have had success with our method on many datasets, we have seen failures as we described in our analysis of the dataset on whole blood measured on 450 k. This raises the issue of when and why the method fails. In recent work, we studied colon cancer and EBV transformation of lymphocytes using WGBS [22, 23]. In these two systems, we observed global hypomethylation as well as an increased variation in global methylation levels in colon cancer and EBV-transformed lymphocytes compared to normal-matched samples from the same person. However, we saw minimal variation in global methylation between three normal samples in both systems. This type of observation is the same as what we see for the scWGBS data on mESCs and MII cells (Fig. 17e); there is substantial heterogeneity in global methylation for mESCs and not for MII cells where the method fails. The same observation is reflected in Fig. 9 where we, as expected, see a substantial variation in cancer, EBV-transformed lymphocytes and cultured fibroblasts, and substantially less variation in samples from whole blood. However, our method does work on normal prostates, which also show minimal variation in global methylation, suggesting that this is not the explanation for the failure. More work is needed to establish firmly whether this ranking holds true for most primary tissues or might be a consequence of oncogenesis, manipulation in culture or a kind of unappreciated batch effect, affecting a well-defined compartment of the genome. We note that the cause of the ranking does not matter; as long as the ranking is present, it can be exploited to reconstruct A/B compartments.

The functional implications of A/B compartments have not been comprehensively described; we know they are associated with open and closed chromatin [1], replication timing domains [6, 35] and changes during mammalian development, and are somewhat associated with gene expression changes [8]. Our work makes it possible to study more comprehensively A/B compartments, especially in primary samples. We have illustrated this with a brief analysis of the relationship between A/B compartments and somatic mutation rate in PRAD.

## Materials and methods

### Infinium HumanMethylation450 BeadChip

We use the standard formula *β*=*M*/(*M*+*U*+100) for estimating percentage methylation given (un)methylation intensities *U* and *M*. Traditionally, the term *M* value is used for the logit transform of the beta value, and we do the same.

With respect to CpG density, the 450 k array probes fall into four categories that are related to CpG islands. CpG island probes (30.9 *%* of the array) are probes located in CpG islands, shore probes (23.1 *%*) are probes within 2 kb of CpG islands, and shelf probes (9.7 *%*) are probes between 2 kb and 4 kb from CpG islands. Open sea probes (36.3 *%*) are the rest of the probes. We use the term CpG resort probes to refer to the union of island, shore and shelf probes; in other words non-open sea probes.

### Methylation data

Methylation data are given in Table 5.

**Table 5:** Methylation data sources

Dataset	Cell type	Number, *n*	Platform	Accession number	Reference
450 k-fibroblast	Fibroblast (skin)	62	450k	[GEO:GSE52025]	[19]
450 k-EBV	LCL (EBV)	288	450k	[GEO:GSE36369]	[17]
450 k-blood	Whole blood	305	450k	[GEO:GSE54882]	[24]
27 k-EBV Vancouver	LCL (EBV)	180	27k	[GEO:GSE27146]	[36]
27 k-EBV London	LCL (EBV)	77	27k	[GEO:GSE26133]	[37]
450 k-PRAD-normal	Prostate (normal)	49	450 k	TCGA	[14]
450 k-PRAD-cancer	Prostate (cancer)	340	450 k	TCGA	[14]
PMDs-IMR90	Fibroblast (lung)	1	MethylC-Seq	Salk Institute	[40]
EBV hypomethylation blocks	LCL (EBV)	1	WGBS	[GEO:GSE49629]	[23]

**The 450 k-fibroblast dataset** The study contains 62 samples from primary skin fibroblasts from [19]. The raw data (IDAT files) are available on GEO under the accession number [GEO:GSE52025].

**The 450 k-EBV dataset** The study contains 288 samples from EBV-transformed lymphoblastoids cell lines (LCL) [17] from three HapMap populations: 96 African-American, 96 Han Chinese-American and 96 Caucasian. The data are available on GEO under the accession number [GEO:GSE36369].

**The 450 k-blood dataset** The study contains 305 samples from whole blood [24]. The data are available on GEO under the accession number [GEO:GSE54882].

**The 27 k-EBV Vancouver dataset** The study contains 180 samples from EBV-transformed LCLs [36] from two HapMap populations: 90 individuals from Northern European ancestry (CEU), and 90 individuals from Yoruban (West African) ancestry (YRI). The processed data are available on GEO under the accession number [GEO:GSE27146].

**The 27 k-EBV London dataset** The study contains 77 EBV-transformed LCLs assayed in duplicates [37]. Individuals are from the Yoruba HapMap population, and 60 of them are also part of the 27 k-EBV Vancouver dataset. The raw data (IDAT files) are available on GEO under the accession number [GEO:GSE26133].

**The 450 k-PRAD-normal and 450 k-PRAD-cancer datasets** At the time of download, the dataset contained 340 PRAD cancer samples from TCGA [14] along with 49 matched normal samples. We used the Level 1 data (IDAT files) available through the TCGA Data portal [38].

**The PMDs-IMR90 dataset** The PMD boundaries from IMR90 [39] are available at [40].

**The EBV hypomethylation blocks dataset** Hypomethylated blocks between EBV-transformed and quiescent B cells were obtained from a previous study [23]. Only blocks with a family-wise error rate equal to 0 were retained (see the reference). The data are available on GEO under the accession number [GEO:GSE49629].

### Processing of the methylation data

For the 450 k-fibroblast and 450 k-PRAD datasets, we downloaded the IDAT files containing the raw intensities. We read the data into R using the illuminaio package [41]. For data normalization, we use the minfi package [26] to apply the Noob background subtraction and dye-bias correction [42] followed by functional normalization [25]. We have previously shown [25] that functional normalization is an adequate between-array normalization when global methylation differences are expected between individuals. For the 450 k-EBV dataset, only the methylated and unmethylated intensities were available, and therefore we did not apply any normalization. For the 450 k-blood dataset, data were quantile normalized and then adjusted for estimated cell proportions and sex as described in [24]. For the 27 k-EBV London dataset, IDAT files were available, and we applied the Noob background correction and dye-bias correction as implemented in the methylumi package [42]. For the 27 k-EBV Vancouver dataset, IDAT files were not available and therefore we used the provided quantile normalized data as discussed in [36].

For quality control of the samples, we used the packages minfi and shinyMethyl [26, 43] to investigate the different control probes and potential batch effects. All arrays in all datasets passed the quality control. After normalization of the 450 k array, we removed 17,302 loci that contain a single-nucleotide polymorphism (SNP) with an annotated minor allele frequency greater than or equal to 1 *%* in the CpG site itself or in the single-base extension site. We used the UCSC Common SNPs table based on dbSNP 137. The table is included in the minfi package.

For the analysis of the 27 k array data, we only considered probes that are also part of the 450 k array platform (25,978 probes retained in total) and applied the same probe filtering as discussed above.

### Construction of 450 k correlation matrices

For each chromosome, we start with a *p*×*n* methylation matrix *M* of *p* normalized and filtered loci and *n* samples. We use *M* values as methylation measures. We compute the *p*×*p* matrix of pairwise probe correlations *C*=cor(*M*^′^), and further bin the correlation matrix *C* at a predefined resolution *k* by taking the median correlation for between CpGs contained in each of two bins. Because of the probe design of the 450 k array, some of the bins along the chromosome do not contain any probes; these bins are removed. As discussed in “Results and discussion,” the correlations of the open sea probes are the most predictive probes for A/B compartments, and therefore the correlation matrix is computed using only those probes (36.3 *%* of the probes on the 450 k array). The inter-chromosomal correlations are computed similarly.

### Hi-C data

Samples are described in Table 6.

**Table 6:** Hi-C data sources

Dataset	Cell	Cell	Accession	Reference
	line	type	number	
HiC-EBV-2009	GM06990	LCL (EBV)	[GEO:GSE18199]	[1]
HiC-EBV-2013	GM12878	LCL (EBV)	[GEO:GSE48592]	[46]
HiC-EBV-2014	GM12878	LCL (EBV)	[GEO:GSE63525]	[7]
HiC-IMR90-2013	IMR90	Fibroblast (lung)	[GEO:GSE43070]	[4]
HiC-IMR90-2014	IMR90	Fibroblast (lung)	[GEO:GSE63525]	[7]
HiC-fibro-skin	–	Fibroblast (skin)	[GEO:GSE41763]	[45]
HiC-fibro-HFF1	HFF-1	Fibroblast (skin)	E-MTAB-1948	[5]
HiC-K562-2009	K562	Leukemia	[GEO:GSE18199]	[1]
HiC-K562-2014	K562	Leukemia	[GEO:GSE63525]	[7]
HiC-mESC-2012	J1	mESC	[GEO:GSE35156]	[3]

### Processing of the Hi-C data

For the datasets HiC-EBV-2014, HiC-K562-2014 and HiC-IMR90-2014 from [7], we used the raw observed contact matrices that were constructed from all read pairs that map to the human genome hg19 with a MAPQ ≥30. These contact matrices are available in the supplementary files of the GEO deposition [GEO:GSE63525]. For the HiC-IMR90-2013 dataset from [4], we used the online deposited non-redundant read pairs that were mapped with Bowtie [44] to human genome hg18 using only the first 36 bases. For the HiC-EBV-2009 and HiC-K562-2009 datasets from Lieberman-Aiden et al. [1], we used the mapped reads deposited on GEO under the accession number [GEO:GSE18199]. Reads were mapped to human genome hg18 using Maq, as described. For the fibro-skin dataset from [45], we merged the reads from two individuals with normal cells (father and age-matched control). We used the processed reads of the GEO deposition [GEO:GSE41763] that were mapped using Bowtie2 to the hg18 genome in an iterative procedure called ICE previously described in [15]. For the HiC-mESC-2012 dataset, we used the mapped reads deposited on GEO under the accession number [GEO:GSE35156]; reads were mapped to the mm9 genome.

For the HiC-EBV-2013 dataset from [46] and the HiC-fibro-HFF1 dataset from [5], we downloaded the SRA experiments containing the FASTQ files of the raw reads. We mapped each end of the paired reads separately using Bowtie to the hg18 genome with the --best mode enabled. We kept only paired reads with both ends mapping to the genome.

For all datasets but the Hi-C datasets from [7], we used the liftOver tool from UCSC to lift the reads to the human genome hg19 version for consistency with the 450 k array. Reads from [7] were already mapped to the hg19 genome.

### Construction of Hi-C matrices

As a first step, we build for each chromosome an observed contact matrix *C* at resolution *k* whose (*i*,*j*)th entry contains the number of paired-end reads with one end mapping to the *i*th bin and the other end mapping to the *j*th bin. The size of the bins depends on the chosen resolution *k*. We remove genomic bins with low coverage, defined as bins with a total count of reads less than 10 *%* of the total number of reads in the matrix divided by the number of genomic bins. This filtering also ensures that low mappability regions are removed.

To correct for coverage and unknown sources of biases, we implemented the iterative correction procedure called ICE [15] in R. This procedure forces bins to have the same experimental visibility. We apply the normalization procedure on a chromosome basis and noted that for each Hi-C dataset, the iterative normalization converged in less than 50 iterations. To estimate A/B compartments, we further normalize the genome contact matrix by the observed–expected procedure [1], where each band of the matrix is divided by the mean of the band. This procedure accounts for spatial decay of the contact matrix.

### DNase-seq data

DNase-seq data sources are listed in Table 7.

**Table 7:** DNase-seq data sources

Dataset	Cell	Number, *n*	Accession	Reference
	line		number	
DNase-EBV	LCL (EBV)	70	[GEO:GSE31388]	[30]
DNase-IMR90	Fibroblast (lung)	4	[GEO:GSE18927]	[31]

**The DNase-EBV dataset** The study contains 70 biological replicates of EBV-transformed LCLs [30] from the HapMap Yoruba population. The data are deposited on GEO under the accession number [GEO:GSE31388] and raw files are available at [47].

**The DNase-IMR90 dataset** The dataset is composed of four technical replicates of the IMR90 fetal lung fibroblast cell line available on GEO under the accession number [GEO:GSE18927].

### Processing of the DNase-Seq data and construction of the correlation matrices

For the DNase-EBV dataset from [30], we downloaded the raw reads in the HDf5 format for both the forward and reverse strands. We converted the reads to bedGraph, lifted the reads to the hg19 genome and converted the files to bigWig files using the UCSC tools. For the DNase-IMR90 dataset, we used the raw data already provided in the bigWig format. Reads were mapped to the hg19 genome. For both datasets, data were read into R using the rtracklayer package [48]. To adjust for library size, we normalized each sample by dividing the DNase score by the total number of reads. For each sample, we constructed a normalized DNase signal at resolution 100 kb by taking the integral of the coverage vector in each bin. This was done using BigWig files and the rtracklayer package in R [48]. All DNase datasets have the same read length within experiment (EBV/IMR90). This results in a *p*×*n* signal data matrix where *p* is the number of bins for the chromosome and *n* the number of samples. We defined the average DNase signal as the across-sample mean of the signal matrix. The DNase correlation matrix is the *p*×*p* Pearson correlation matrix of the signal matrix.

### GC content correction of the DNase data

For GC content correction of the DNase data, we fitted a LOESS curve of the DNase signal against the bin GC content for each sample differently and regressed out the fitted relationship.

### scATAC-seq data

scATAC-seq data were obtained from GEO under the accession number [GEO:GSE68103] described in [13]; see Table 8. We used data processed by the authors, specifically the file GSM1647124_CtlSet1.dhsmatrix.txt.gz. This experiment represents data on a mixture of two cell lines: GM12878 and HL60. We use the data processed by the authors of the paper, which consist of a matrix of accessibility across 195,882 known hypersensitive sites (from ENCODE) and 4538 cells. Each hypersensitive site is furthermore characterized as being specific to GM12878, specific to HL60 or common across the two cell types. To classify each cell to a cell type, we computed the total number of reads in each of the cell-type-specific hypersensitive sites. This yields two numbers per cell. These numbers are further normalized by (1) the total number of reads in all hypersensitive sites scaled to 2000 reads (slightly more than the median number of reads per cell) and (2) the number of cell-type-specific hypersensitive sites scaled to 50,000 sites. The final scale is the number of reads mapped for a cell with a read depth of 2000 and a cell type with 50,000 hypersensitive sites. These numbers are displayed in Fig. 16a. Cells are assigned to the GM12878 cell type if they have more than three times as many normalized reads for this cell type, compared to HL60; in other words if they are below the *y*=*x*/3 line in the figure. Subsequently we discarded hypersensitive sites that had no reads in any of the cells and obtained 631 bins at a resolution of 100 kb on chromosome 14. Eigenvectors were computed and smoothed as described below.

**Table 8:** Single-cell epigenetic data sources

Dataset	Cell type	Organism	Number, *n*	Accession number	Reference
scATAC-EBV	LCL (EBV)	Human	2679	[GEO:GSE68103]	[13]
scWGBS-mESC	mESC	Mouse	20	[GEO:GSE56879]	[12]
scWGBS-MII	MII	Mouse	12	[GEO:GSE56879]	[12]

### scWGBS data

scWGBS data were obtained from GEO under the accession number [GEO:GSE56879] described in [12]; see Table 8. We used data processed by the authors, specifically the files GSM1370555_Ser_X.CpG.txt.gz where X takes values 1 to 20. These files describe the single CpG methylation levels of 20 individual cells for mESCs cultured in serum conditions. We removed CpGs within 4 kb of a CpG Island (using the CpG Islands defined in [49]), as we did for the 450 k methylation array data. We next binned the genome in 100-kb bins and computed, for each bin, the average methylation value across all CpGs in the bin. Bins with a total coverage of less than 100 were removed from the analysis. This resulted in a binned methylation matrix, which was used to compute an empirical correlation matrix. Eigenvectors were computed and smoothed as described below.

### Eigenvector analysis

To obtain eigenvectors of the different matrices from Hi-C, DNA methylation and DNase data, we use the non-linear iterative partial least squares (NIPALS) algorithm implemented in the mixOmics package in R [50]. Each eigenvector is smoothed by a moving average with a three-bin window, with the following exceptions. For the 450 k data, we used two iterations of the moving average smoother. For the single-cell epigenetic data, we used a window size of five bins with two iterations of the moving average smoother for ATAC-seq and three iterations for WGBS.

When we compare eigenvectors from two different types of data, we only consider bins that exist in both data types; some bins are filtered out in a data-type-dependent manner, for example, because of the absence of probes or low coverage. This operation slightly reduces the number of bins we consider in each comparison.

Because the sign of the eigenvector is arbitrarily defined, we use the following procedure to define a consistent sign across different chromosomes, datasets and data types. For Hi-C data and DNase data, we correlate the resulting eigenvector with the eigenvector from Lieberman-Aiden et al. [1], changing sign if necessary to ensure a positive correlation. For DNA methylation data, we use that the long-range correlations are significantly higher for the closed–closed interactions. We therefore ensure that the eigenvector has a positive correlation with the column sums of the binned correlation matrix, changing sign if necessary. This procedure results in positive values of the eigenvector being associated with closed chromatin and the B compartment as defined in Lieberman-Aiden et al. [1] (in this paper they ensure that negative values are associated with the closed compartment).

To measure the similarity between two eigenvectors, we use two measures: correlation and compartment agreement. The correlation measure is the Pearson correlation between the smoothed eigenvectors. The compartment agreement is defined as the percentage of bins that have the same eigenvector sign, interpreted as the percentage of bins that belong to the same genome compartment (A or B) as predicted by the two eigenvectors. Occasionally, this agreement is restricted to bins with an absolute eigenvector value greater than 0.01 to discard uncertain bins.

Because open chromatin regions have a very high DNase signal in comparison to closed chromatin regions, the DNase signal distribution is highly skewed to the right; therefore, we center both the average signal and the first eigenvector by subtracting their respective medians, before computing the correlation and agreement.

### Somatic mutations in PRAD

We obtained a list of somatic mutations in PRAD from the TCGA data portal [38]. Several lists exist; we used the Broad Institute curated list: broad.mit.edu__IlluminaGA_curated_DNA_sequencing_level2.maf. To obtain capture regions, we queried the CGHub website [51] and found that all samples were profiled using the same capture design described in the file whole_exome_agilent_1.1_refseq_plus_3_boosters.targetIntervals.be obtained from the CGHub bitbucket account.

Somatic mutation rates in each 100-kb genomic bin were computed as the number of mutations inside each bin, divided by the length of the capture regions inside the bin.

### Data

Estimated compartments for TCGA cancer data are available in Additional file 2. We processed 450 k IDAT files from TCGA with Noob [42] followed by functional normalization [25] as implemented in the minfi [26] package. Compartments were estimated using compartments() of minfi version 1.15.11.

### Software

Software for performing the analysis of 450 k methylation arrays described in this manuscript have been added to the minfi package [26] version 1.15.11 or greater, available through the Bioconductor project [52, 53]. The main function is compartments(). A script implementing our method for DNase-seq is available as Additional file 3.

## Additional files

Additional file 1
**Supplementary Figures.** Figures S1–S15. (PDF 2088 kb)

Additional file 2
**TCGA A/B compartments.** Estimated compartments for 12 TCGA cancers using 450 k methylation data, as a concatenated TXT file. There are six columns; the file is tab separated and has a header. Column 1 (tcga_code) is the TCGA short code for the cancer. Columns 2 (chr), 3 (start) and 4 (end) are the chromosomal coordinates of each region, in hg19 coordinates (1-based) and at 100-kb resolution. Column 5 (eigen) is the estimated first eigenvector. Column 6 (domain) is whether or not the region is estimated to be in open or closed chromatin. (TXT 13619 kb)

Additional file 3
**Code for DNase-seq data.** Software for our method for DNase-seq data in the form of an R script. (ZIP 2 kb)

## Abbreviations

ATAC: assay for transposase-accessible chromatin
BLAC: bladder urothelial carcinoma
BRCA: breast invasive carcinoma
ChIP: chromatin immunoprecipitation
COAD: colon adenocarcinoma
DNase: deoxyribonuclease
EBV: Epstein–
Barr virus; ENCODE: Encyclopedia of DNA Elements
GEO: Gene Expression Omnibus
HNSC: head and neck squamous cell carcinoma
ICE: iterative correction and eigenvector decomposition
KIRC: kidney renal clear cell carcinoma
KIRP: kidney renal papillary cell carcinoma
LCL: lymphoblastoid cell line
LIHC: liver hepatocellular carcinoma
LUAD: lung adenocarcinoma
LUSC: lung squamous cell carcinoma
MII: metaphase II
mESC: mouse embryonic stem cell
NIPALS: non-linear iterative partial least squares
PMD: partially methylated domain
PRAD: prostate adenocarcinoma
scATAC: single-cell assay for transposase-accessible chromatin
scWGBS: single-cell whole-genome bisulfite sequencing
seq: sequencing
SNP: single-nucleotide polymorphism
TCGA: The Cancer Genome Atlas
UCEC: uterine corpus endometrial carcinoma
WGBS: whole-genome bisulfite sequencing
