# Supplementary material for: Reconstructing A/B compartments as revealed by Hi-C using long-range correlations in epigenetic data
Source: Genome Biol. 2015 Aug 28;16(1):180. doi: 10.1186/s13059-015-0741-y (PMC4574526; doi:10.1186/s13059-015-0741-y)
Supplement: Additional file 1 — Supplementary Figures. Figures S1–S15. (PDF 2088 kb) [file 13059_2015_741_MOESM1_ESM.pdf]

# Additional file 1: Supplementary Figures for Reconstructing A/B compartments as revealed by Hi-C using long-range correlations in epigenetic data

Jean-Philippe Fortin[1] and Kasper D. Hansen[1,2,*]

[1]Department of Biostatistics, Johns Hopkins Bloomberg School of Public Health
[2]McKusick-Nathans Institute of Genetic Medicine, Johns Hopkins School of Medicine

*To whom correspondence should be addressed. Email: khansen@jhsph.edu

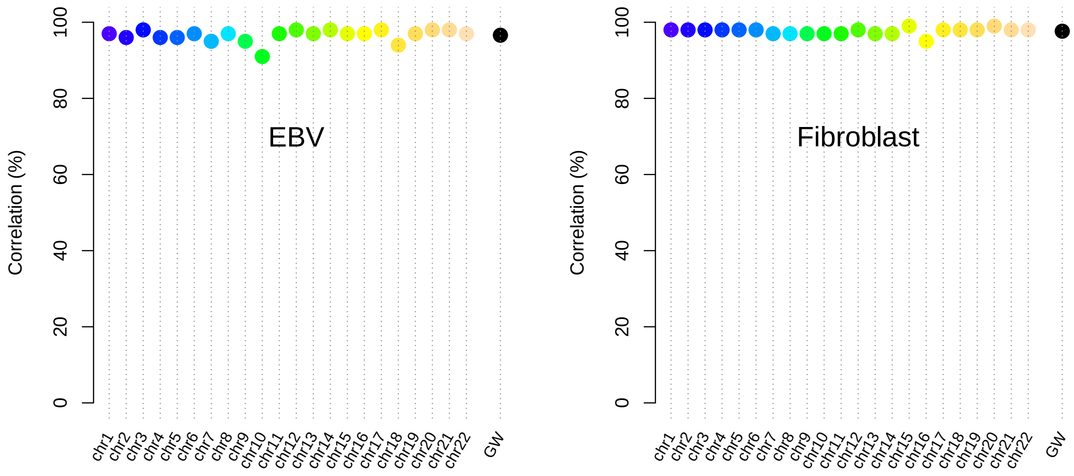


**(a)**

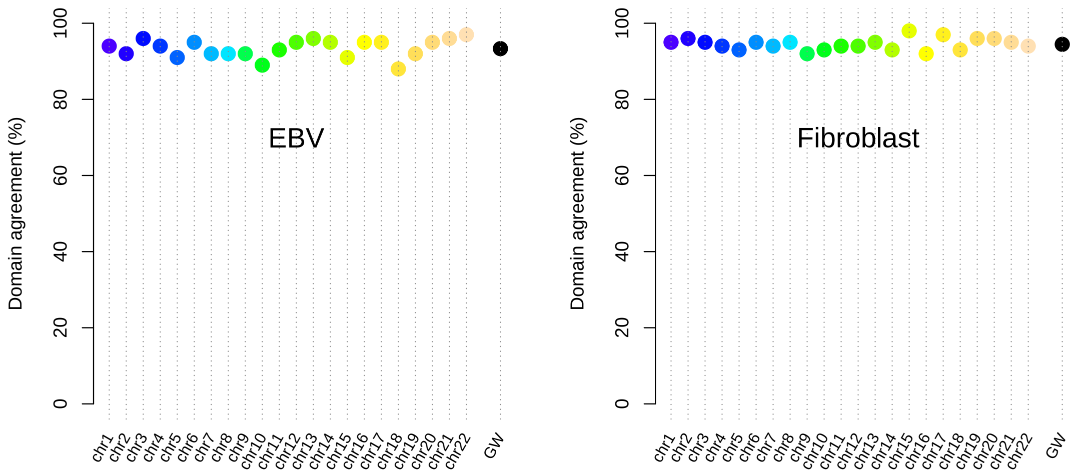


**(b)**

**Figure S1. Correlations and domain agreement between eigenvectors obtained from independent Hi-C experiments.** (a) For each chromosome, and for the whole genome, we report the correlation between the eigenvectors of two different experiments from the same cell type at 100kb resolution. The two different experiments are HiC-EBV-2014 vs. HiC-EBV-2013 (EBV) and HiC-IMR90-2014 vs. HiC-IMR90-2013 (Fibroblast). (b) Like (a), but using domain agreement as similarity measure.

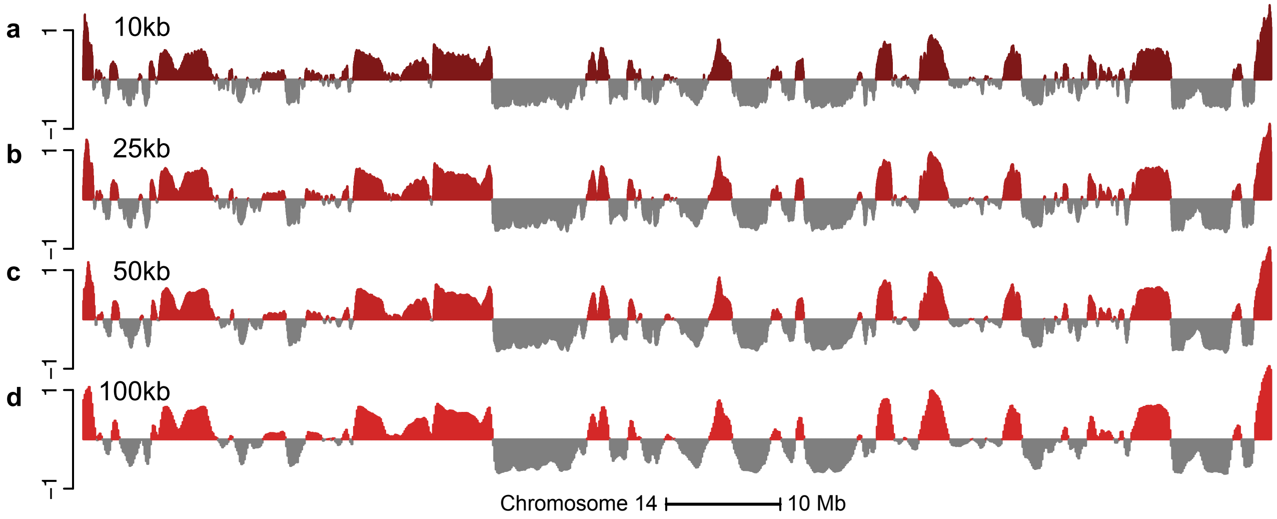


**Figure S2. A/B compartments revealed by Hi-C data do not change at resolutions higher than 100kb.** The figure displays data on all of chromosome 14 at different resolutions. The four different tracks represent the first eigenvector of the HiC-IMR90-2014 dataset at resolutions (a) 10kb, (b) 25kb, (c) 50kb and (d) 100kb.

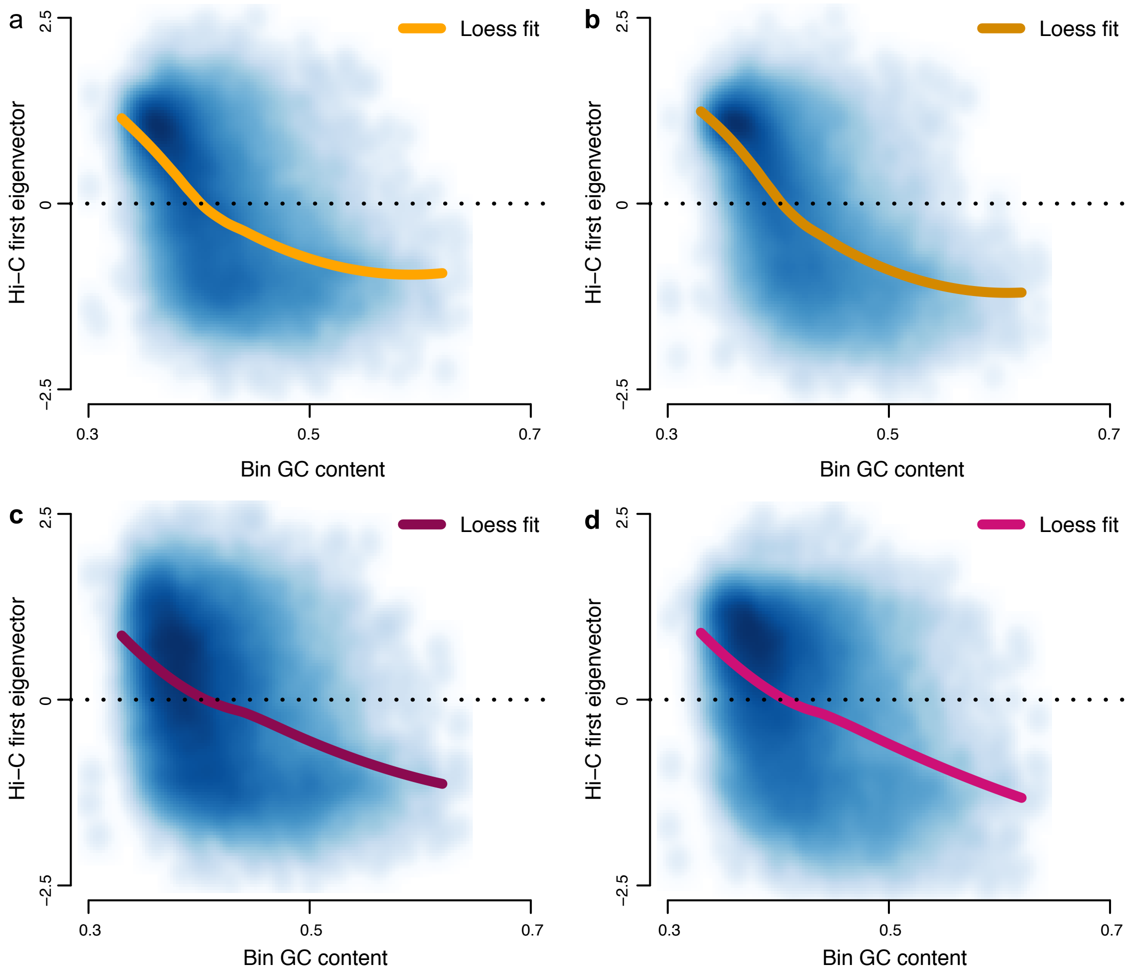


**Figure S3. Association between the Hi-C eigenvectors and GC content is reproducible and cell-type specific.** The four plots represent the genome-wide eigenvector value for each bin against the GC content of the bin at resolution 100kb. The four datasets are (a) HiC-EBV-2014, (b) HiC-EBV-2013, (c) HiC-IMR90-2014, and (d) HiC-IMR90-2013. Note that the experiments (a) and (c) are from the same laboratory, while the experiments (b) and (d) are from different labs. The orange lines represent the loess fit for GC content. We observe that loess curves from the same cell type (the two at the top, and then the two at the bottom) are more similar to each other than across cell types, despite different protocols and experiment years. This reflects the cell-type specificity of the GC content association with the Hi-C first eigenvector, reflecting a functional association rather than a pure technical bias.

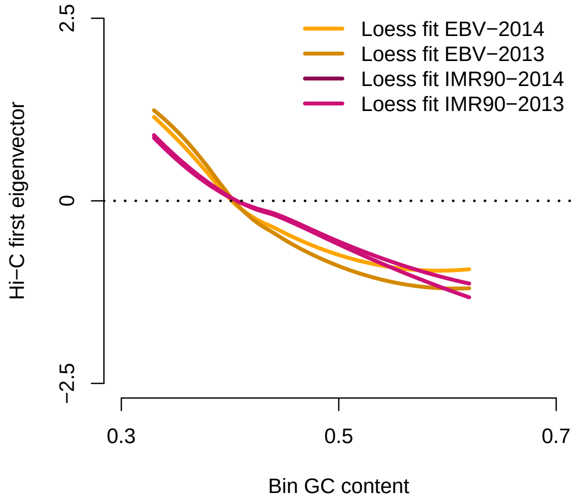


**(a)**

**Figure S4. Association between the Hi-C eigenvectors and GC content is reproducible and cell-type specific.** The four loess fits come from Figure S3. The relationship between the Hi-C eigenvectors and GC content is cell-type specific.

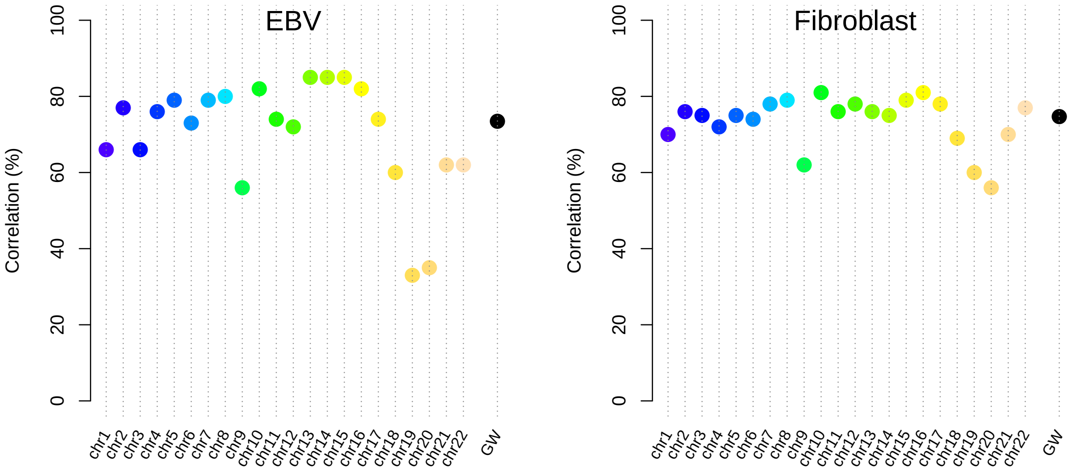


**(a)**

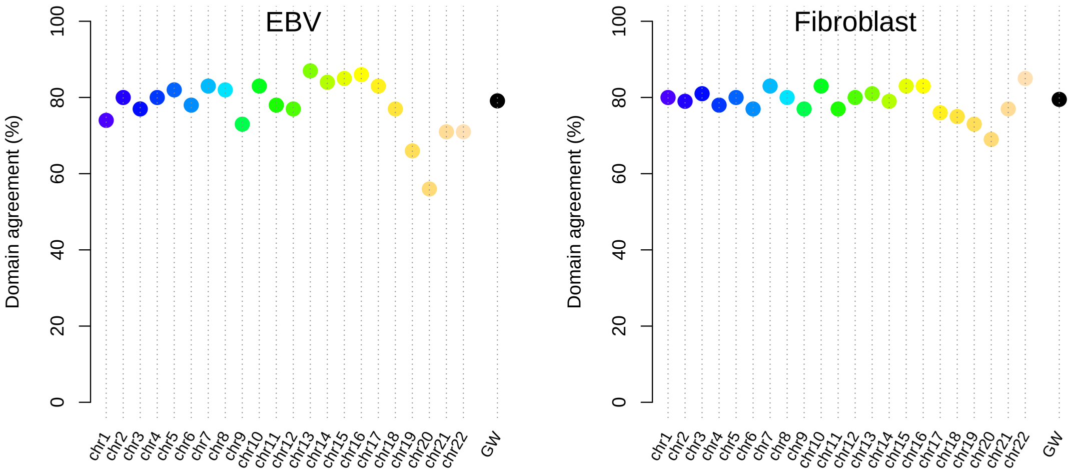


**(b)**

**Figure S5. Correlations and domain agreement between eigenvectors obtained from Hi-C and 450k experiments.** (a) For each chromosome, and for the whole genome, we report the correlation between the eigenvectors obtained from Hi-C and 450k experiments on the same cell type, specifically we compare HiC-EBV-2014 to 450k-EBV (EBV) and HiC-IMR90-2014 to 450k-Fibroblast (Fibroblast). (b) Like (a), but using domain agreement as similarity measure.

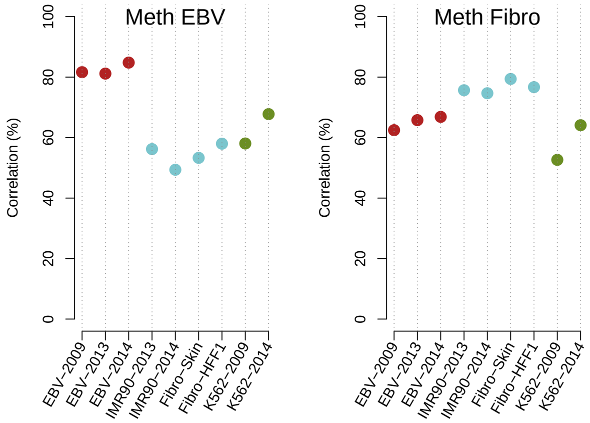


**Figure S6. Compartment predictions based on 450k data are cell-type specific.** Correlations between the eigenvectors from 9 different Hi-C datasets across 3 different cell types with both the 450k-EBV and 450k-Fibroblast datasets. We observe higher correlation between Hi-C data and DNA methylation data when the comparison is being made for the same cell type.

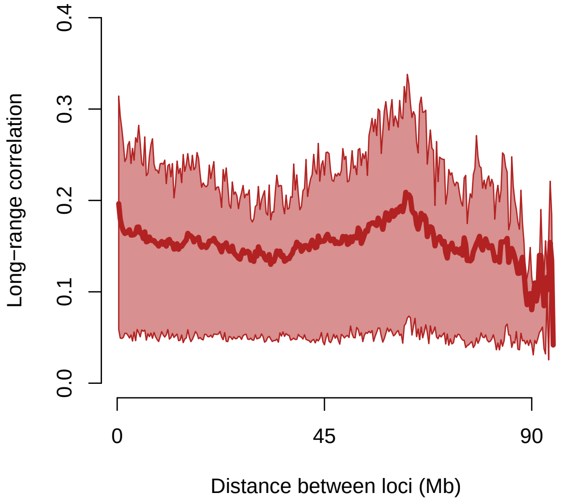


**(a)**

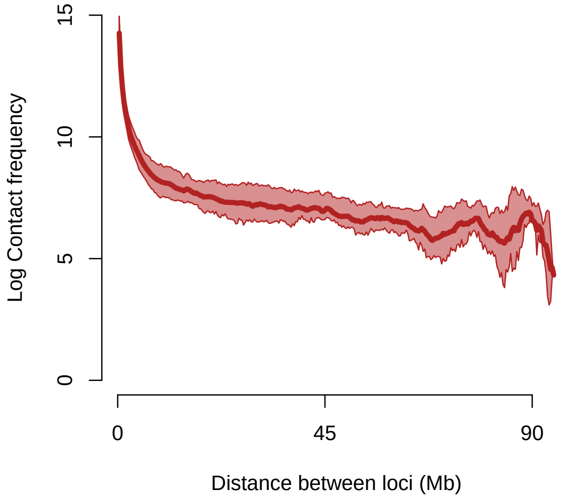


**(b)**

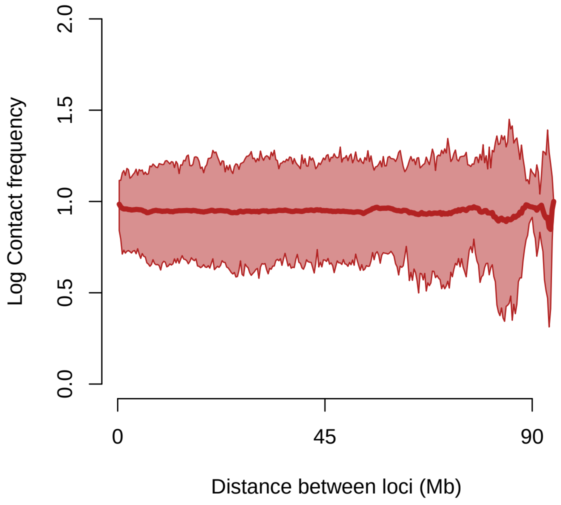


**(c)**

**Figure S7. Correlation as function of distance between loci.** In each figure we display the median and 25%,75%-quantiles of the entries in a given matrix as a function of genomic distance. (a) Entries in the binned correlation matrix of the 450k-EBV dataset. (b) Entries in the ICE normalized log contact matrix for the HiC-EBV-2014 experiment. (c) As (b) but the matrix has been normalized using the expected-observed method.

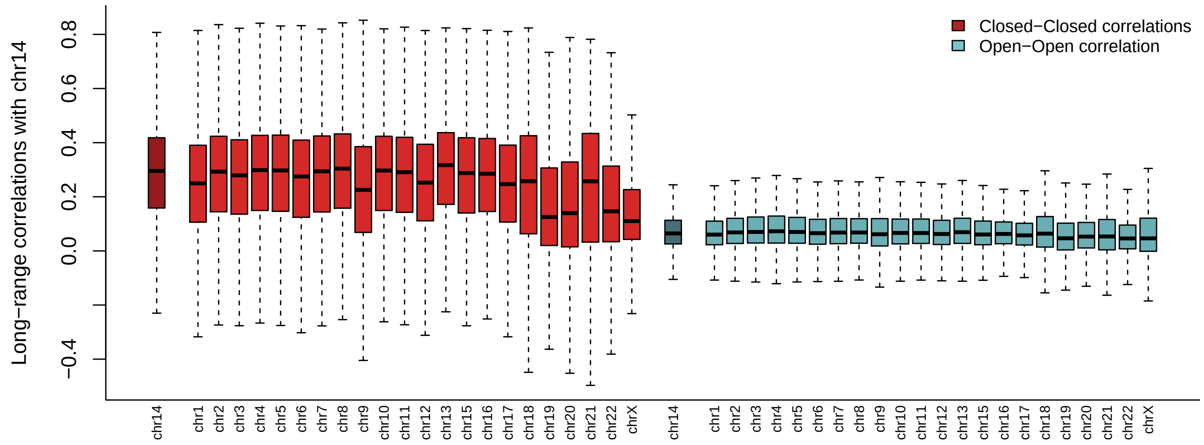


**Figure S8. Between-chromosome correlations of DNA methylation.** Each boxplot shows the binned correlations between bins on chromosome 14 and bins on other chromosomes for the 450k-EBV dataset. The boxplots are stratified by whether the correlation is inside the open compartment or inside the closed compartment (open to close compartment correlations are not depicted). The open and closed compartments were defined using the HiC-EBV-2014 dataset.

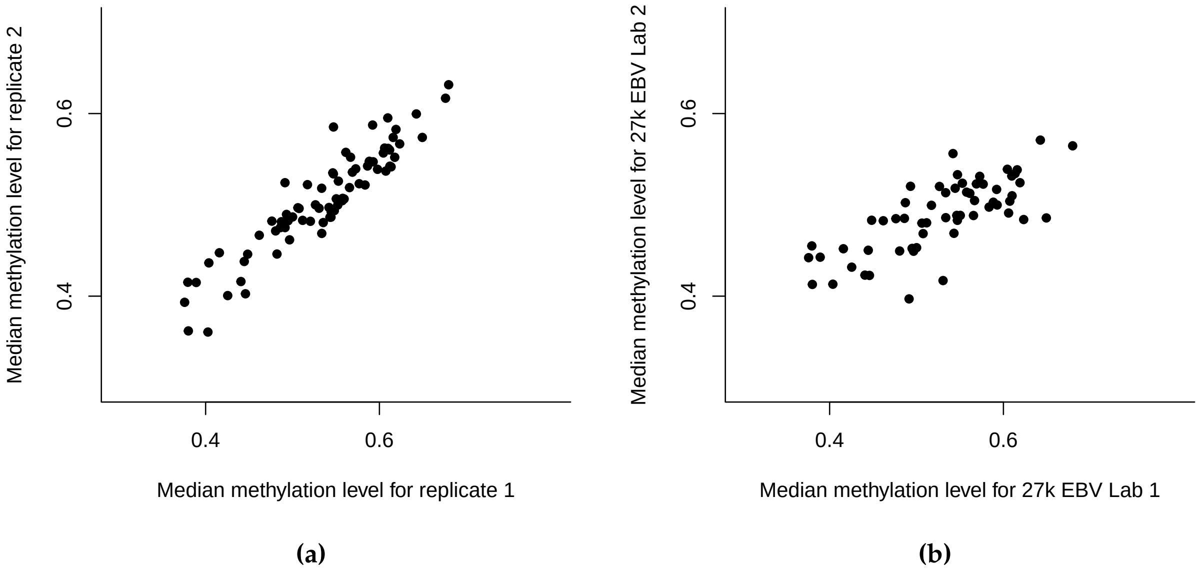


**Figure S9. Sample ranking based on methylation levels in the closed compartments replicate across experiments.** We computed the average methylation level of open sea probes in the closed compartment. The compartments were defined using the HiC-EBV-2014 data. (a) Comparison between hybridization replicates from the 27k-London dataset. (b) Comparison between the same samples assayed in two different experiments, the 27k-Vancouver and the 27k-London experiments.

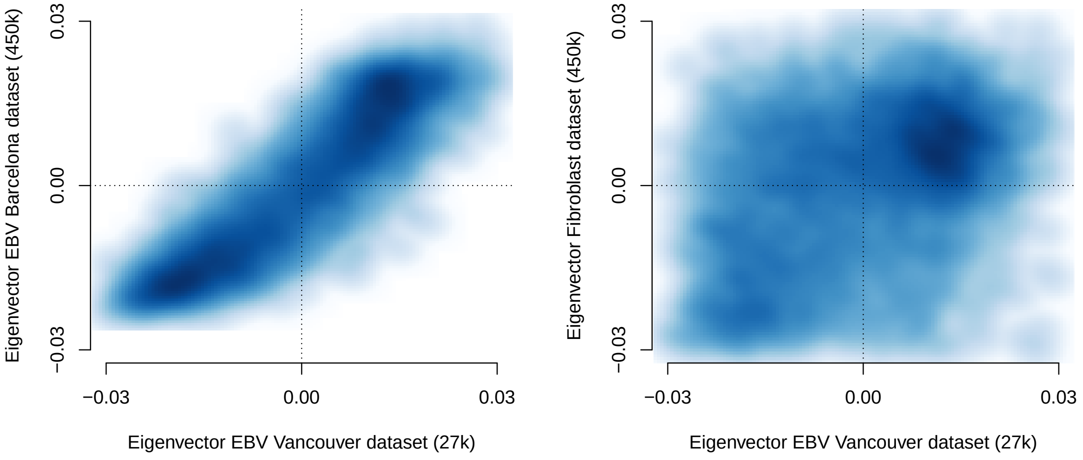


**Figure S10. Validation of the 450k EBV eigenvector using a 27k dataset.** The eigenvector for the 27k dataset was computed using the full (no binning) correlation matrix of all 5599 autosomal probes located in open sea which are common between the two platforms. (a) A comparison between eigenvectors from the 27k-Vancouver dataset and the 450k-EBV dataset. The correlation between the two eigenvectors is 89.3%. (b) A comparison between eigenvectors from the 27k-Vancouver dataset and the 450k-Fibroblast dataset. The correlation is 42.1%, confirming the cell-type specificity of the methylation eigenvector.

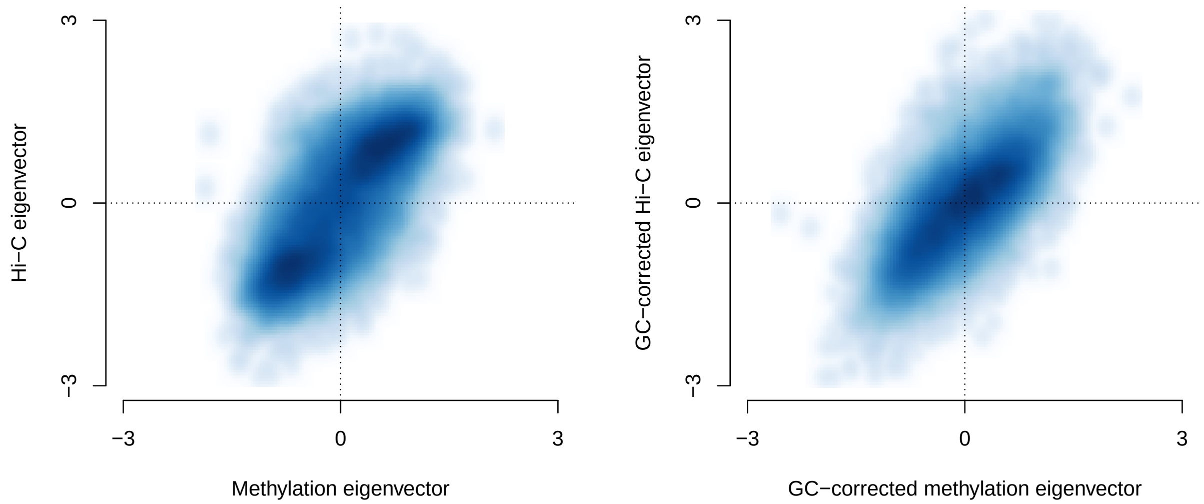


**Figure S11. Effect of GC content adjustment on the Hi-C and methylation eigenvectors.** (a) Genome-wide eigenvector for the HiC-EBV-2014 dataset against the eigenvector for the 450k-EBV dataset before GC content loess adjustment. (b) Same as (a) but after GC content adjustment using loess correction. Adjusting the two eigenvectors for GC content by using loess regression does not remove the good correlation between the two datatype eigenvectors.

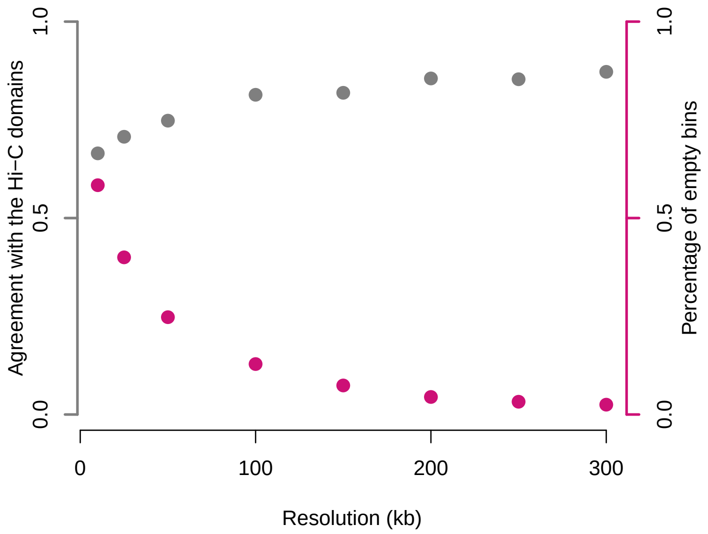


**Figure S12. Performance of the A/B compartment prediction for different binning resolutions of the 450k array.** The grey points depict the increase in agreement between compartments estimated using the 450k-EBV and HiC-EBV-2014 datasets as binning resolutions coarsen. The pink points show the increase in empty bins as binning resolution is increased. We conclude that a resolution of 100kb is a good choice.

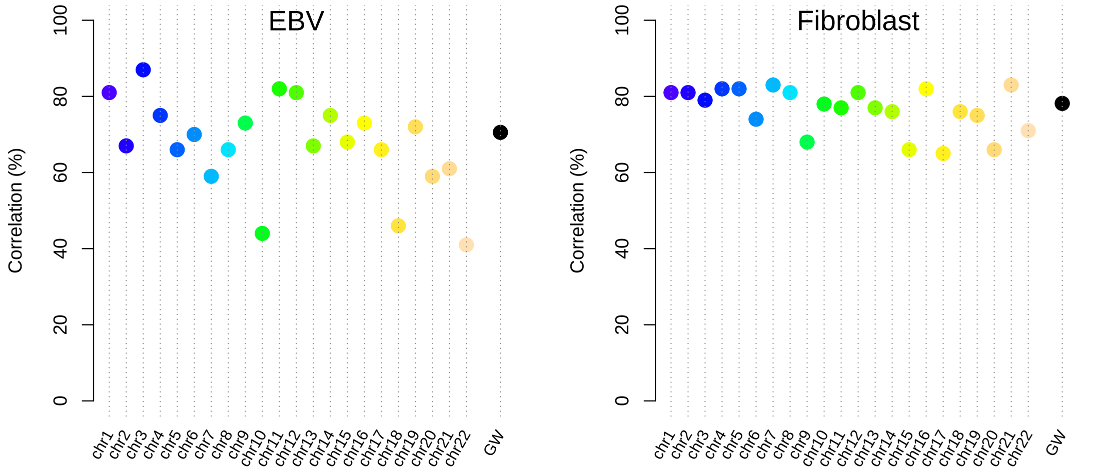


**(a)**

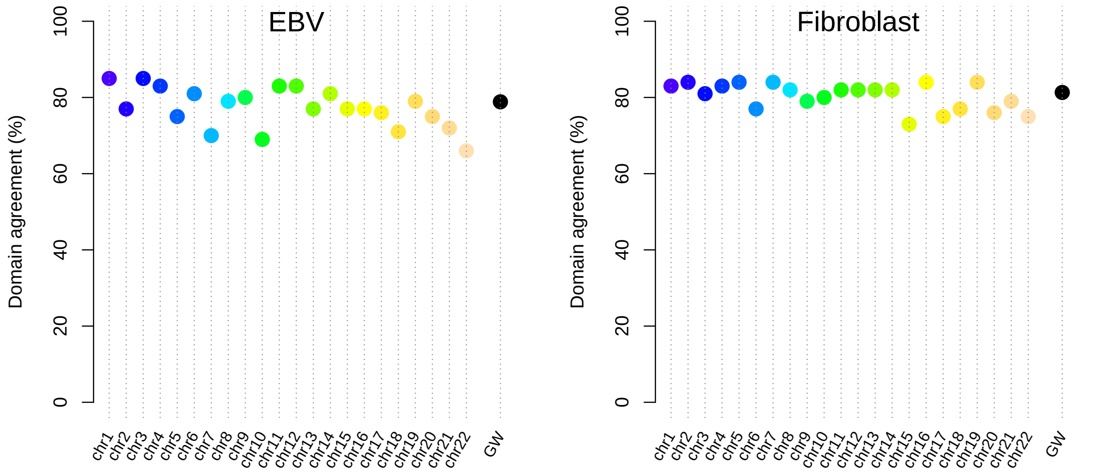


**(b)**

**Figure S13. Correlations and domain agreement between Hi-C eigenvectors obtained from Hi-C and DNase experiments.** (a) For each chromosome, and for the whole genome, we report the correlation between the eigenvectors obtained from Hi-C and DNase experiments on the same cell type, specifically we compare the HiC-EBV-2014 to DNase-EBV dataset (EBV) and HiC-IMR90-2014 to DNase-IMR90 (Fibroblast). (b) Like (a), but using domain agreement as similarity measure.

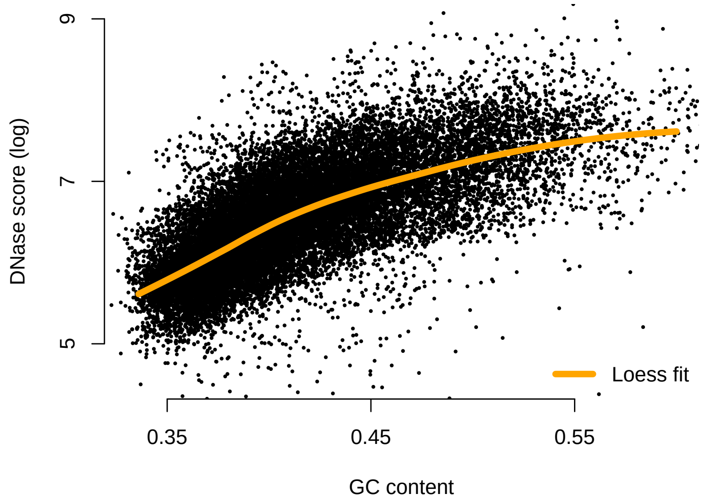


**(a)**

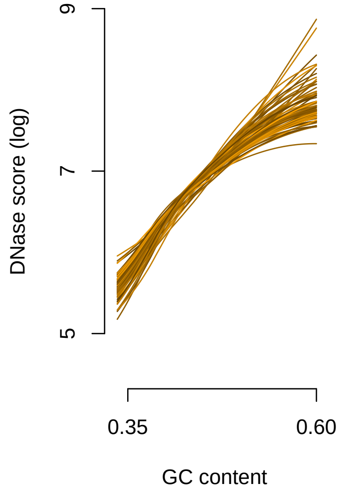


**(b)**

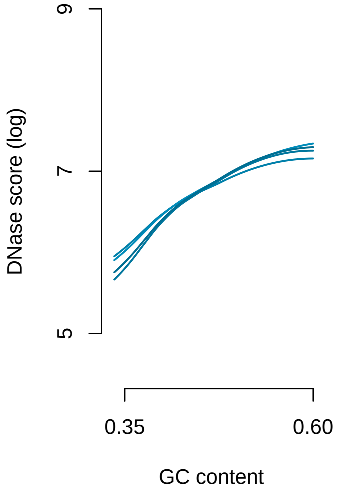


**(c)**

**Figure S14. Relationship between DNase scores and GC content.** (a) Relationship between the log DNase score of one individual from the DNase-EBV dataset and GC content at the bin level, genome-wide (100kb resolution); loess fit is in orange. (b) Loess fits, as described in (a), for all 70 individuals from the DNase-EBV dataset (c) Loess fits for 4 replicates from the IMR90 cell line (DNase-IMR90 dataset)

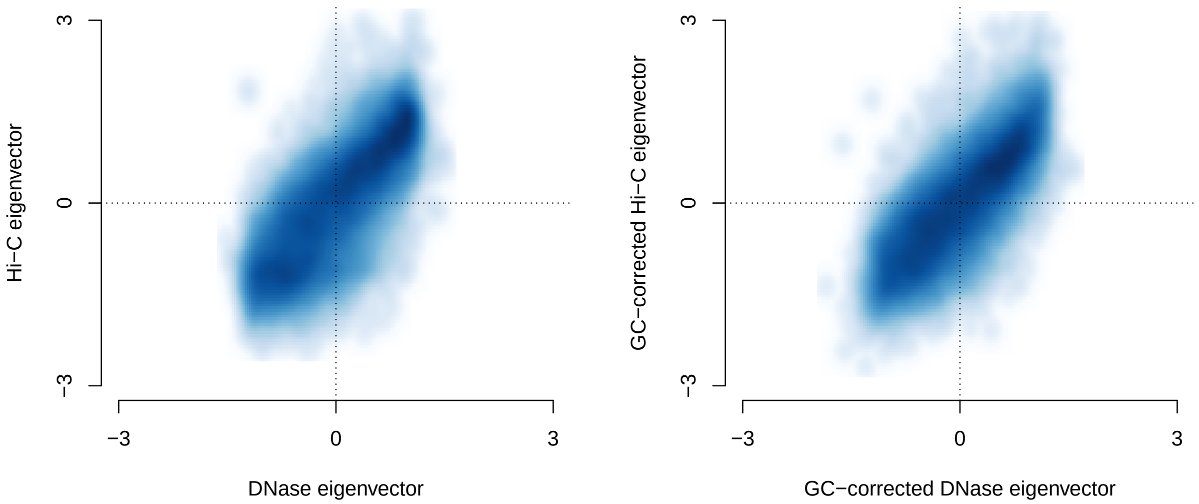


**Figure S15. Effect of GC content adjustment on the Hi-C and DNase eigenvectors.** (a) Genome-wide eigenvector for the HiC-IMR90-2014 dataset against the eigenvector for the DNase-IMR90 dataset before GC content loess adjustment. (b) Same as (a) but after GC content adjustment using loess correction. Adjusting the two eigenvectors for GC content by using loess regression does not remove the good correlation between the two datatype eigenvectors.
